# On the occasion of the centennial of insulin therapy (1922–2022), II-Organotherapy of diabetes mellitus (1906–1923): Acomatol. Pancreina. Insulin.

**DOI:** 10.1007/s00592-022-02014-7

**Published:** 2022-12-31

**Authors:** Alberto de Leiva-Hidalgo, Alejandra de Leiva-Pérez

**Affiliations:** 1 Universidad Autónoma de Barcelona, Barcelona, Spain; 2 Fundación DIABEM., Barcelona, Spain

**Keywords:** Pancreatic extracts, Organotherapy, Acomatol, Pancreina, Insulin, Patents

## Abstract

**Aims:**

The general objective has been the historiographical investigation of the organotherapy of diabetes mellitus between 1906 and 1923 in its scientific, social and political dimensions, with special emphasis on the most relevant contributions of researchers and institutions and on the controversies generated on the priority of the "discovery" of antidiabetic hormone.

**Methods:**

We have analyzed the experimental procedures and determination of biological parameters used by researchers during the investigated period (1906–1923): pancreatic ablation techniques, induction of acinar atrophy with preservation of pancreatic islets, preparation of pancreatic extracts (PE) with antidiabetic activity, clinical chemistry procedures (glycemia, glycosuria, ketonemia, ketonuria, etc.). The field investigation has included on-site and online visits to cities, towns, buildings, laboratories, universities, museums and research centers where the reported events took place, obtaining documents, photographic images, audiovisual recordings, as well as personal interviews complementary to the documentation consulted (primary sources, critical bibliography, reference works). The documentary archival sources have been classified according to theme, including those consulted in situ with those extracted online and digitized copies received mainly by email. Among the many archives contacted, those listed below have been most useful and have been consulted on site and on repeated visits: National Library of Medicine-Historical Archives (Bethesda, MD, USA); Archives, University of Toronto and Thomas Fisher Rare Books Library (Toronto, Ontario, Canada); Francis A. County Library of Medicine, Harvard University (Boston, Mass, USA); Zentralbibliothek der Humboldt-Universität (Berlin, DE), Geheimarchiv des Preuβischen Staates (Berlin, DE); Landesamt für Bürger—und Ordnungsangelegenheiten (LABO) (Berlin, DE); Arhivele Academiei Române şi Universitǎții Carol Davila (Bucharest, RO).

**Main results and conclusions:**

A) The European researchers Zülzer (Z Exp Path Ther 23:307–318, 1908) and Paulescu (CR Seances Soc Biol Fil 85:558, 1921) meet the requirements of the priority rule in the discovery of the antidiabetic hormone. B) Factors of socioeconomic and political nature related with the First World War and the inter-war period delayed the process of purification of the antidiabetic hormone in Europe. C) The Canadian scientist J. Collip, University of Alberta, temporarily assimilated to the University of Toronto, and the American chemist and researcher G. Walden, with the expert collaboration of Eli Lilly & Co., were the main authors of the purification process of the antidiabetic hormone. D) The scientific evidence, reflected in the heuristics of this research, allows to assert that the basic investigation carried out by the Department of Physiology of the University of Toronto, directed by the Scottish J. Macleod, in conjunction with the clinical research undertaken by the Department of Medicine of the University of Toronto (W. Campbell, A. Fletcher, D. Graham) made it possible in record time the successful treatment of patients with what was until then a deadly disease.

## Georg Ludwig Zülzer (1870–1949): Acomatol

Georg Ludwig Zülzer (also spelled Zuelzer) was born in 1870 in Berlin, and studied in Freiburg and Berlin, where he defended his doctoral thesis in 1893 at the Friedrich Wilhelm University (now Humboldt University) [1]. He furthered his postgraduate training working as an assistant with Raphaël Lépine and Léon Bouveret in Lyon, with Carl H. von Noorden, Albert Neisser and Hermann Eichhorst in Frankfurt am Main and with Franz Riegel in Gießen, where he also met Ferdinand Blum, who would become a collaborator and a friend. In 1900, Zülzer started a private practice as an internist in Berlin.

### Zülzer's experiences with pancreatic extracts in experimental diabetes

In 1903, influenced by Blum’s description of adrenergic diabetes [2], Zülzer began experimental studies with pancreatic and adrenal extracts, using the rabbit as an experimental animal. During the first years, he used for his experiments the laboratory of the institute headed by Professor T. W. Engelmann in Berlin.

Zülzer was the first to demonstrate that urinary glucose excretion following adrenalin injection correlated with an elevation of blood glucose levels [3], and noted that the simultaneous administration of a pancreatic extract (PE) prevented the hyperglycaemic effect of adrenalin. He measured the potency of the extract by the amount needed to neutralize the hyperglycemia caused by the administration of one milligram of adrenalin [4].

This spurred his interest in investigating the effects of equine, bovine and porcine pancreatic extracts on glycemia and glycosuria in the pancreatectomized dog. In total, Zülzer devoted twelve years (from 1902 to 1914) to researching the biological effects of PE. His work resulted in seven publications and three patents [5].

Initially, the experiments were financed by Chemische Fabrik Auf Actien (formerly E. Schering, now Bayer HealthCare Pharmaceuticals), that provided Zülzer with the help of laboratory technicians Max Dohrn and Anton Marxer. As an extraction medium, Zülzer used alcohol, which resulted in the precipitation of a large number of proteins. However, he did not succeed in removing all contaminating proteins. (To attain a higher degree of purification of the extract, it is necessary to use fractional precipitation with increasing concentrations of alcohol). To achieve a complete separation of the proteins, Zülzer heated the filtrate and added sodium chloride. The last step in the extraction procedure was to vaporize the alcoholic filtrate under vacuum; the residue obtained in powder form contained the active substance [5].

In these experiments Zülzer macerated glandular tissue in mortar on sand, mixed it with silica gel and pressed it on a tissue filter; he treated the liquid extract with alcohol, precipitating contaminating proteins. The experiments were carried out in 1905, but only published in 1908. The first one was performed in August. The dog weighed 8 kg. Diet consisted of 300 g of meat and 100 g of oil per day. After intravenous administration of 5 ml of the preparation (containing 1 g of the extract) a reduction in daily glycosuria from 28.6 to 19 g was recorded [6].

### Treatment of eight patients with pancreatic extracts

Between 1906 and 1908, Zülzer was able to treat eight patients with diabetes from different hospitals with his PE. They showed a certain degree of clinical improvement and a reduction in glycosuria and ketonuria, although often accompanied by side effects such as fever, sweating, vomiting, stomatitis and muscle hypertonia [7].

**Fig. 1:**
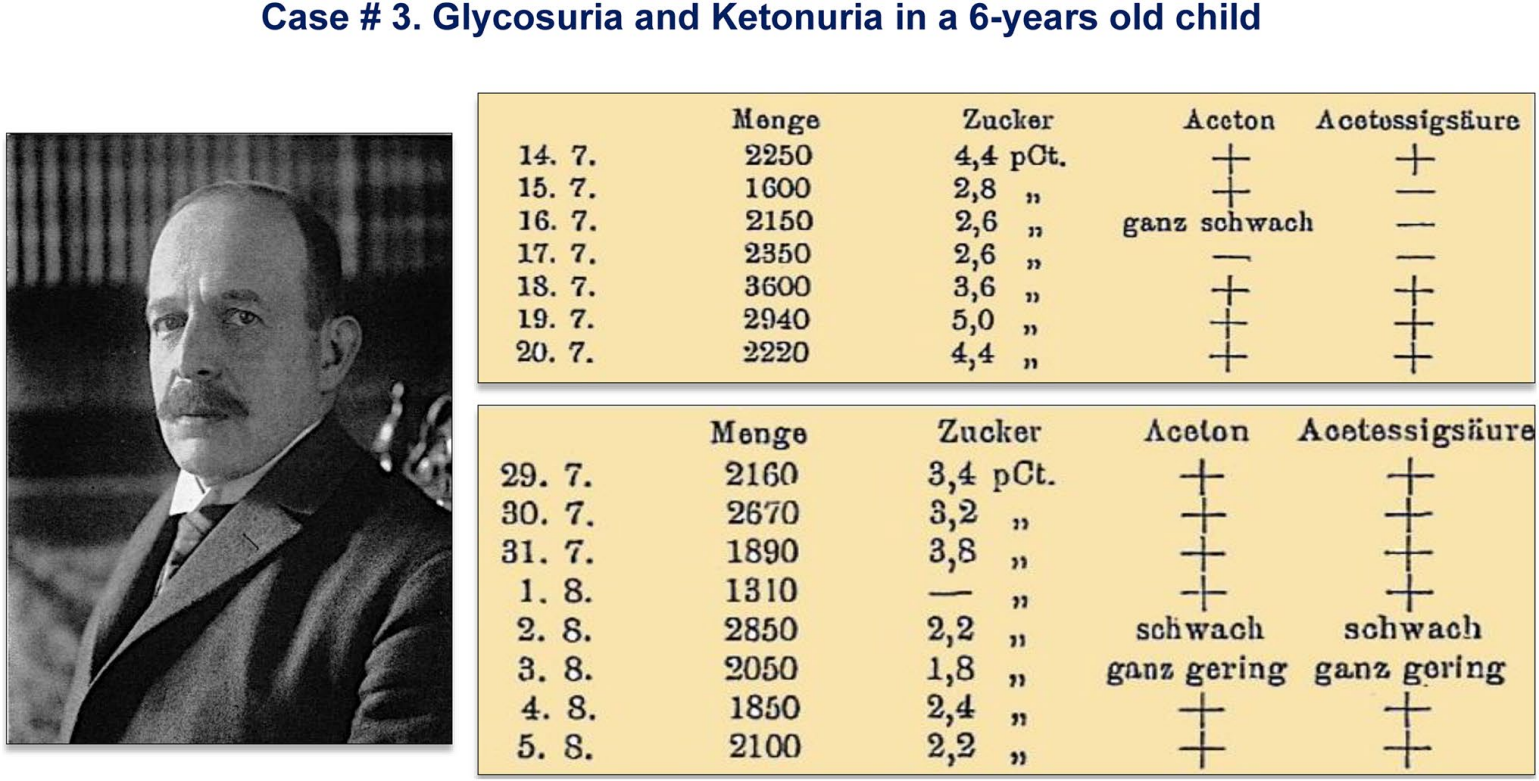
Photography of Georg L. Zülzer; unknown author and date. Historical Archive, Humboldt University, Berlin. Public domain. Case 3: A 6-year-old boy with severe diabetes, ketonuria, heavy glycosuria and malnutrition. Effect of the pancreatic extract [7]

One special case was a 6-year-old boy with severe diabetes, ketosis, heavy glycosuria and malnutrition. On July 14, 1907, he received intravenously an emulsion containing 1 g of PE. Body temperature rose immediately after the injection to 38.4 °C, with associated vomits. On July 16, his clinical condition improved; the boy gained weight, ketones disappeared and glycosuria decreased (Fig. 1). On August 1, after the injection of 1 g of PE, a new peak of fever developed (39.2 °C) but glucose excretion decreased and ketone bodies disappeared again. After 48–72 h without available extract, the clinical and chemical parameters deteriorated. Zülzer could not produce new doses; the boy was discharged and died some days later.

Zülzer presented his results at a meeting of specialists in Internal Medicine held on 15 June 1908 in Berlin, and received favorable comments in a landmark textbook on endocrinology written by Professor Arthur Biedl of the University of Vienna [8].

### Obtaining patents for the pancreatic extract: Acomatol

**Fig. 2:**
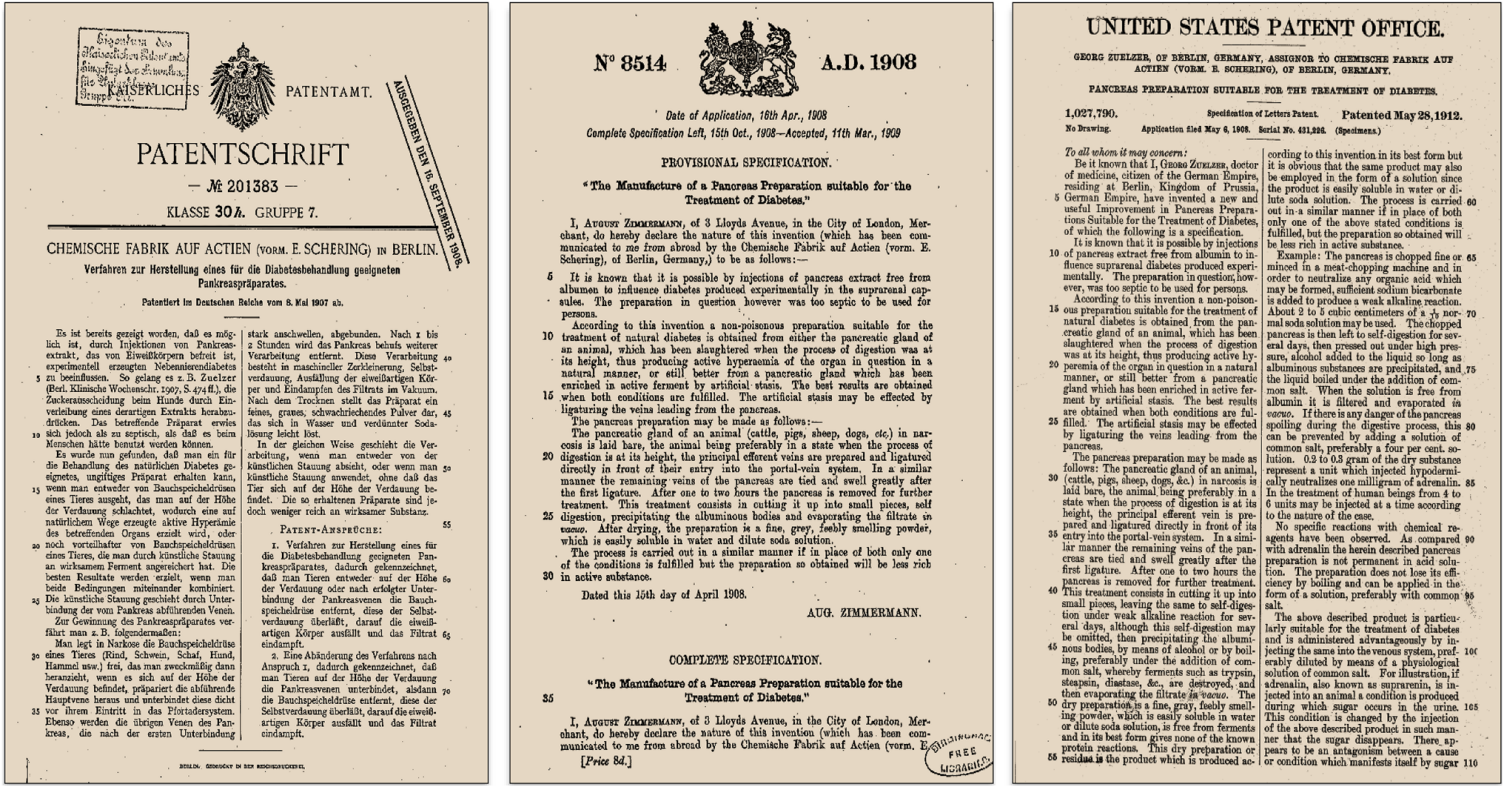
G.L. Zülzer. German (1908), British (1909) and USA (1912) patents for Acomatol. Public domain

On May 8, 1907, Chemische Fabrik auf Actien applied for a patent of Zülzer’s extract (Acomatol) to the German Patent Office (Kaiserliches Patentamt). Patent number 201383 was granted on September 20, 1908, for a “pancreatic extract preparation to be used to control severe hyperglycemia and diabetic coma”. On April 16, 1908, an application was filed to the Great Britain Patent Office. On March 11, 1909, Patent 8514 was granted for the “manufacture of a pancreas preparation suitable for the treatment of diabetes” On May 6, 1908, a new application was filed to the United States Patent Office. USA patent 1,027,790 was granted on May 28, 1912 (Fig. 2) [9].

In 1909, J. Forschbach reproduced Zülzer’s experiments in Minkowski’s laboratory in Breslau, with three dogs and three patients. Glycosuria decreased and Forschbach noted that “Zülzer was the first to produce, successfully, from the pancreas, a preparation that eliminates sugar excretion in a shorter or longer period by intravenous administration”. However, Forschbach ceased human experimentation after observing high fever and other toxic effects in two patients [10]. As a consequence, Chemische Fabrik auf Actien decided to withdraw its investment to the research.

### Collaboration between Georg Zülzer and Camille Reuter

In 1911, Zülzer, who in 1908 had been appointed Chief Physician at Hasenheide Hospital in Berlin, signed an agreement with the Swiss pharmaceutical company Hoffmann-La Roche (now Roche) to further research on pancreatic extracts. With its financial support, Zülzer was able to set up an experimental laboratory at Hasenheide Hospital. Camille Reuter, chemical engineer working in Hoffmann-La Roche’s research laboratory in Grenzach (Baden-Württemberg), joined the project.

**Fig. 3:**
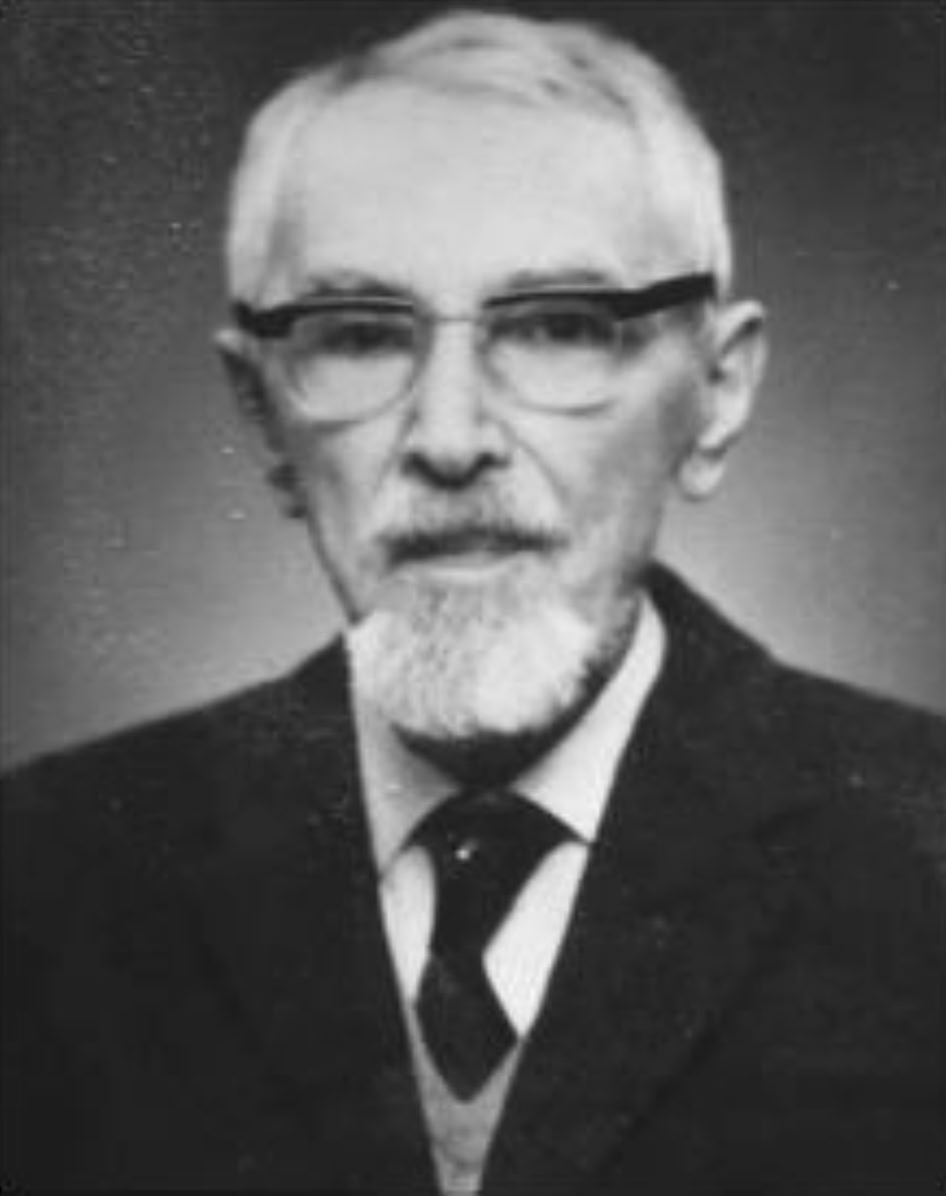
Portrait of Camille Reuter. Unknown date and author. (Courtesy of V. Jörgens)

Reuter’s main tasks were to purify the extract and to boost the manufacture at large scale following a new protocol. He used the hydraulic press and the Buchner funnel in the process of elaborating the new pancreatic extract in collaboration with Zülzer (Fig. 3) [11]:

**Table:** 

1- To grind the fresh horse’s pancreas with sand after the addition of sodium bicarbonate, mixing the product with kieselguhr [*]
2- To apply a pressure of 350 atmospheres in a hydraulic press, collecting the filtrate in a Buchner system
3- To mix the fluid with alcohol and filter off the sediment
4- To evaporate the filtrate at low temperature in a vacuum, add water and neutralize the solution
5- To heat the solution (80 °C), perform a dialysis, filter the content with a sterilized Berkefeld-Donde filter and dilute the final preparation of the extract in 0.45 NaCl

**Table:** 

Volumes of 15–50 ml of the pancreatic extract were slowly administered to dogs intravenously. Blood samples (5 mL) were collected at intervals. A significant number of dogs developed severe convulsions. In 1914 when blood glucose was measured by a newly introduced micro-method, convulsions were directly related to hypoglycemia. Blood glucose decreased by 50% or even further in most dogs (in one animal it went down to 17 mg/dL, before dying of hypoglycemia). Acute toxicity and other side effects were not present in these experiments. Control experiments (precipitation of the pancreatic extract with lead acetate) gave negative results. Similar results were achieved with glandular extracts from different animal sources [11, 12]	

[*kieselguhr: diatomaceous earth].

Until the introduction of the first blood glucose micro-determinations in 1913 by Ivar Bang [13], previous procedures had major disadvantages, such as the need for prior separation of proteins from the sample and a laborious protocol that required two days to complete blood glucose quantification [14, 15]. The publication of the Hagedorn-Jensen micro-method would still take another five years to develop [16].

Before the end of 1914, the laboratory at Grenzach had processed over 100 kg of animal pancreas. To maintain the blood glucose lowering-effect, the pancreatic extract had to be administered at intervals of 3–4 h. In August 1914, Zülzer’s research was interrupted since he was called to serve as a military doctor in World War I. Hoffmann-La Roche cancelled the project, concerned about the war, the potential risks of hypoglycemia and the need for close surveillance of the patients. After the war, Zülzer returned to Berlin in 1918, weakened by malaria. He became Chief of Medicine at Lankwitz Hospital, a municipal institution of the Union of Health Insurance Companies in Berlin (Verband der Krankenkassen), with no affiliation to the university.

### Georg L. Zülzer claimed his priority right on the discovery of the antidiabetic hormone. Other scientists’ views on Zülzer's activities and the relevance of Acomatol

In the article that described the clinical cases treated with insulin by the University of Toronto research team, published in the *British Medical Journal* in January 1923, the authors referred to Zülzer's previous research, stating that in 1908 he treated six diabetic patients in whom he obtained a reduction and even a disappearance of glycosuria and ketonuria by intravenous administration of the PE of his invention; however, due to the extreme lability of the extract and the serious side effects, Zülzer's preparation was not used in the clinical setting [17].

On October 16th, 1923, Zülzer circulated, through the international press agent Transatlantic, a typewritten message entitled "The overcoming of diabetes. New facts about insulin", in which he claimed his priority right on the discovery of Acomatol, a preparation that was successfully used to treat severe forms of diabetes, including coma, years ahead of insulin [18].

**Table:** 

(…) Without intending to deny the painstaking efforts of Dr. Banting or to lessen his merits for the medical science I venture to say that the actual discovery of the preparation has been made by me a decade ago under the name of Acomatol
(…) The preparation is able to reduce the content of saccharine matter in the diabetic human body is an hitherto unheard of degree, relieving the patient even from the most severe form of diabetes, the so-called coma diabeticorum
(…) Acomatol was completed after prolonged studies in my laboratories. The quantity production should just be arranged as the World War began. Insulin shows all the characteristic qualities of my Acomatol
(…) It was already in the year 1908 that I have cured the first strongly diabetic persons with Acomatol
(…) The preparation showed then still some technical imperfections which however were eliminated in 1914

Zülzer expressed his regret at the news that the 1923 Nobel Prize in Physiology or Medicine had been awarded to J.J.R. Macleod and F.G. Banting of the University of Toronto. He claimed his priority right on the discovery of the antidiabetic hormone in an article published in the German journal *Medizinische Klinik*, in which he criticized the fact that his work had been neglected by the German institutions [19].

### J.J.R. Macleod publicly acknowledged Zülzer's research on pancreatic extracts

In a lecture at the 11th International Congress of Physiology in Edinburgh in 1923 [20], J.J.R. Macleod publicly acknowledged Zülzer’s work in developing PE with antidiabetic activity, demonstrating in 1908 the ability of these extracts to neutralize hyperglycemia induced by the administration of epinephrine as well as clinical manifestations of human diabetes mellitus.

Macleod also acknowledged Zülzer in his Nobel Lecture [21]:

**Table:** 

(…) In 1907 Zuelzer published results which must be considered, in the light of what we know now, as really demonstrating the presence of the antidiabetic hormone in alcoholic extracts of pancreas
(…) But unfortunately, even though several diabetic patients were benefited by administration of the extracts, Zuelzer himself was discouraged in continuing them because of toxic reactions in the treated patients

In his book entitled *Carbohydrate Metabolism and Insulin* [22, p. 56], Macleod stated that

**Table:** 

Zuelzer came very near to the discovery of a method for preparing insulin, as we know it today	

Zülzer and Macleod exchanged postal correspondence in October–November 1927.

In 1968, Ian Murray, Professor of Physiology at Anderson College of Medicine and Head of the Department of Metabolic Diseases, University of Glasgow, a founding member of the International Diabetes Federation, initiated an investigation into the priority of antidiabetic hormone isolation, after which he asserted his duty to vindicate the merits of Georg Zülzer as the first to achieve a pancreatic preparation of potential therapeutic value [23].

### Criticisms of Zülzer

A. Labhart, Department of Medicine, University of Zurich, reviewed in detail in a 1978 publication the experiences of G.L. Zülzer in experimental diabetes and stated: “Zuelzer for 12 years doggedly pursued his idea and yet at the end fell short. He had the substance—the Acomatol, as he called it—in hand, yet he didn't dare to make use of it because of the misinterpretation of its effects” [24]. Fate was against him: lack of available micro-methods for blood glucose determination, envious colleagues, difficulties in his academic promotion, suspension of funding, and worst of all, the outbreak of the Great War, ruined Zülzer’s most promising project. Hasenheide became a military institution and he himself, having joined the army as a doctor, ended his diabetes research.

Zülzer was determined to obtain a high academic position at the University of Berlin, where his father had been Chair in the Department of Internal Medicine. During two research stays in Berlin in April and August 2015, we consulted the existing documentation on Zülzer in the Historical Archives of Humboldt University, and in the Secret Archives of the Prussian State (Geheimes Staatsarchiv Preuβischer Kulturbesitz—GStA PK). The documents in both archives reveal a conflicting situation between Zülzer and the academics responsible for the admission and promotion of professors at the Faculty of Medicine. Between 1919 and 1932, the Medical Faculty voted on several occasions against his nomination as *privatdozent*. Wolf Wilhelm Zülzer (Georg’s son, a paediatric pathologist, director of the Michigan Research Center, Detroit Children's Hospital, as well as Director of the Division of Hematologic, Heart and Lung Diseases at the US National Institutes of Health) noted in 1985: “Medical historians have written that Georg Zülzer was defeated by narrow-mindedness and uncooperativeness of his academic brethren (…). He was a loner by temperament, as well as an offender against accepted dogma” [25].

Finally, on March 19, 1932, the Prussian Minister of Science, Art and Education, Adolf Grimme, assigned him the Chair of Pathology and Therapy of Infectious Diseases, appointment subject to the possibility of revocation [26].

After Hitler's rise to power, Zülzer lost his position at the University, owing to his Jewish origins. Bernhard Rust, the new Minister of Health, Science and Public Education, revoked Zülzer’s teaching license on November 24, 1933 [26]. Zülzer was also expelled from Lankwitz Hospital and all his properties were confiscated. He decided to flee the Nazi regime and in 1934 went to exile to the USA. He worked in the city of New York as a clinician in private practice until his retirement to a nursing home in 1947. Zülzer died on October 15th, 1949, as a consequence of heart failure due to chronic myocarditis. His grave in the White Chapel Memorial Park Cemetery, in Troy (Michigan) bears the following inscription: “The first physician to bring diabetic patients out of terminal coma with his extracted pancreas preparation”.

## Ernest L. Scott (1877–1966)

Ernest Lyman Scott, from the Department of Physiology at Columbia University, was initiated in diabetes research in 1911 as candidate to M. A. His first publication appeared in 1913. Scott suggested that the external digestive enzymes of the pancreas had a deleterious effect on the active principle of the internal secretion of the gland. It was hoped that the presence of these enzymes could be eliminated by the atrophy of the gland which followed complete ligation of the ducts. Nevertheless, after various attempts in the dog, the method was abandoned as impractical [27].

In subsequent work, Scott intended to render inactive these enzymes by the action of highly concentrated ethanol. For this purpose, he elaborated the following protocol:

**Table:** 

The glands were extracted during 72 h at 35–40 °C; then filtered and evaporated to dryness and the residue extracted with ether at room temperature. Once discarded the fraction extracted with ether, the residue was dissolved in 95% ethanol. After a new evaporation process, the extract was diluted in saline solution and ready for injection
Scott also prepared watery extracts. After handling the alcoholic extracts as above, the preparation was evaporated to dryness under a vacuum and extracted in absolute alcohol at 38 °C. After pouring off, the residue was extracted with water rendered acid by acetic acid. Then the preparation was filtered and evaporated. Before injecting, the ethanol was eliminated and saline solution added

In general, the intravenous injections of the PE to pancreatectomized dogs temporarily decreased the sugar excretion. The interpretation suggested by Scott at this point was that the PE could reduce the output of sugar by a toxic action, rather than therapeutic [28].

Scott performed additional experiments with cats. Surprisingly, the administration of PE did not show hypoglycemic effects; it revealed an increase in blood glucose levels by approximately 20%. A logical explanation for this result could not be provided at that time [29].

## John R Murlin (1874–1960)

John Raymon Murlin and Benjamin Kramer, researchers of Cornell University, initially thought that the decreased urinary excretion of glucose, found after the administration of their preparations containing a dual extract of pancreas and duodenal mucosa, was due to changes in renal permeability, rather than to a hormonal effect. Their manuscript was published in the year 1913 [30].

Years later, they noted that the disappearance of glycosuria ran parallel to a decrease of blood sugar levels and concluded that the pancreatic extract actually contained the internally secreted active principle [31].

In 1922, Murlin and coworkers observed that pancreatic extracts caused tissue toxicity with ulceration at the injection sites, resulting in death of some dogs [32].

In cooperation with C. Sutter, Murlin reported the case of a diabetic patient with ketosis treated at the Rochester General Hospital in July 1922 with pancreatic extract administered through a gastrointestinal catheter and by the oral and subcutaneous routes. Only in this latter case could glycosuria and ketonuria be decreased. On July 26, 1922 blood glucose level went down from 513 to 241 mg/dL [33].

## Israel Kleiner (1885–1966)

Israel S. Kleiner and S.J. Meltzer, from the Department of Medical Research at the Rockefeller Institute, investigated the effects of intravenous injection of a pancreas emulsion into intact and depancreatized animals. They reported that in the group of healthy animals, blood glucose concentration levels became equal to those seen before glucose administration at 90 min of the infusion of isotonic glucose. In untreated pancreatectomized animals, however, glucose levels 90 min after glucose administration were more than two times higher than the original value. In a third group of animals, the addition of PE allowed for a very close to normal response. These experiments suggested that internal pancreatic secretion contributed to the rapid disappearance of circulating glucose [34].

In 1919, Kleiner published a set of experiments conducted between 1915 and 1919 supporting the existence of internal pancreas secretion and showing beneficial effects for the treatment of experimental diabetes. Intravenous administration of pancreatic emulsion achieved a highly significant blood glucose reduction in most of the 16 dogs with diabetes after pancreatectomy. Submaxillary gland emulsions (control group) did not change blood glucose levels. No relevant toxic effects occurred. Unfortunately, Kleiner left the Rockefeller Institute and abandoned this area of research [35].

In 2010, Jeffrey M. Friedman carried out a statistical analysis of the raw data presented by I.S. Kleiner. The study reaffirmed the validity of Kleiner's conclusions, confirming the existence of an endocrine pancreatic secretion with antidiabetic action. Unfiltered aqueous pancreatic extract generated from fresh adult pancreas, diluted in physiological saline and slowly administered intravenously was effective in inducing a temporary decrease in blood glucose to normal values and elimination of glycosuria in the dog with post-pancreatectomy diabetes [36].

## Nicolae C. Paulescu: The discovery of pancreina

### Paulescu, outstanding endocrine researcher

Nicolae Constantin Paulescu was born in Bucharest on October 30, 1869. He went to Paris in 1888 for his medical education. In 1891 he obtained a medical post as an extern at the Hôtel-Dieu. In 1897 he defended his doctoral thesis “Recherches sur la structure de la Rate” and was appointed Deputy Surgeon at the Notre Dame du Perpétuel-Sécours Hospital*.* Paulescu entered the Faculty of Science, obtaining the degrees in Biological Chemistry and General Physiology, a doctorate in Natural Sciences [37] and, finally, Doctor from the University of Paris, the Sorbonne (1901) [38]. During these years he was supervised by Étienne Lancereaux and Albert Dastre, with whom he initiated, already in 1889, investigations with the aim to isolate the antidiabetic pancreatic substance [37]. He was introduced in surgical procedures for experimental research and investigated the physiology of the thyroid [39] and adrenal glands [40], the significance of albuminuria [41] and the treatment of arterial aneurysms [42, 43].

Paulescu was offered the post of professor and Chair of Medicine, at the University of Freiburg (Switzerland). Instead, he decided to return to Romania in 1900, where he was appointed Assistant Professor of Physiology and then promoted to Professor of Physiology in 1905 [44].

During the first fifteen years in Bucharest, he organized the laboratory of Experimental Physiology, made important developments in endocrine physiology and experimental surgery and worked as clinician in the Hospital Saint Vincent de Paul.

Paulescu invented an original technique of experimental hypophysectomy, entering the cranial vault laterally, lifting the temporal lobe which allowed optimal visualization of the gland and complete ablation. This procedure was successful in twenty-four experimental cases [45, 46]. The adoption by Harvey Cushing of this new surgical approach resulted in much lower mortality rate in human surgery in comparison with the previous transpharyngeal method, facilitating the “carefold avoidance of the total removal of the anterior lobe” [47, 48]. Lewis Redford, Surgical Fellow working with Cushing, made the following observation: “The best work has been done in Romania by the physiologists Nicolae Paulescu, who has developed what seemed to be an ideal surgical approach to the pituitary of dogs” [49, p. 208]. The inspiration of Paulescu enabled Cushing to carry out extensive animal studies to evaluate the functions of the pituitary gland [50].

Paulescu investigated the glycogenic function of the liver, heart and muscle, in fasting and postprandial stages [51]. Years later, he persued additional research on the metabolism of glycogen in diabetes induced by pancreas ablation [52] and by floridzine [53]. In collaboration with Lancereaux, he developed a four-volume *Traité de Médecine* (3,870 pages). The first volume was printed in 1903, and the three additional ones in 1908, 1912 and 1930, respectively [54]. In different chapters, this comprehensive text covered many aspects of the biology of the pancreatic gland (embryology, anatomy, histology, physiology, clinical assessment, pancreatic disorders from various causes and the influence of carbohydrates, lipids and proteins on glycogen metabolism [37].

### Tecnique and metabolic effects of pancreatectomy

Paulescu was obliged to close the laboratory in 1916, when Bucharest was occupied by Austrian and German troops. All documents he wrote between 1916 and 1919 were published in the following years. One of these documents was the three volumes of the *Traité de Physiologie Médicale* [55], a textbook where he described on pages 313–314 of volume II an original procedure of pancreas ablation he carried out in the experimental laboratory before August 1916.

**Table:** 

“Une condition expérimentale “sine qua non” est que cette ablation soit tout a fait complète. Pour remplir ce postulat, nous avons imaginé un procédé particulier –qui nous a donné entière satisfaction (…).
L’opération dure environ une demi-heure, à savoir: 5 minutes, pour enlever le lobe du foie; 15 minutes, pour extirper le pancréas; 10 minutes, jusqu’à la fin du pansement.
Pour pouvoir opérer dans des bonnes conditions, il faut choisir des chiens jeunes, qui pèsent de 8 à 12 kg, car ils ont tissu conjonctif peu résistant et des lobules pancréatiques très friables, qui peuvent être facilement déchiquetés. De plus, chez ces animaux, l’extrémité splénique de la glande n’est pas trop profondément située et on peut l’enlever, sans grande peine”.

English translation:

**Table:** 

A "sine qua non" experimental condition is that this ablation is complete. To fulfil this postulate, we have devised a particular procedure which has given us complete satisfaction (...).
The operation lasts about half an hour, namely: 5 minutes to remove the liver lobe; 15 minutes to remove the pancreas; 10 minutes until the end of the dressing.
To be able to operate in good conditions, it is necessary to choose young dogs, weighing between 8 and 12 kg, because they have weak connective tissue and very friable pancreatic lobules, which can be easily shredded. In addition, in these animals, the splenic extremity of the gland is not too deeply located and can be removed without much difficulty.

Paulescu demonstrated the requisite of complete ablation to induce experimental diabetes: “Total extirpation of the pancreas is immediately followed by an intense and very grave diabetes…partial ablation of the pancreas does not bring about diabetes if the remaining fragment is greater than a tenth of the gland”. He also observed that ligature of the pancreatic ducts did not induce glycosuria, neither the surgical removal of the duodenal region of the gland [55].

### Development of the pancreatic extract. Intravenous administration to the experimental animal

In pages 321–327 of the *Traité de Physiologie Médicale*, Paulescu described the procedure to develop the PE, followed by four experiments performed in the interval from November 12 to December 29, 1916 [55].

**Table:** 

“Puis, en prenant des précautions minutieuses d’aseptique, on hache cette glande dans un broyeur Latapie, stérilisé au four. Ensuite, on ajoute, à ce hachis, dix fois son poids d’eau distillée stérilisée, et après l’avoir agité à plusieurs reprises, on le place à la glacière.
Au bout de 24 heures, on filtre ce hachis dilué, à travers une double compresse de tarlatane stérili-sée, et on ajoute, au filtrarum, 7 pour 1.000 de NaCl.
Ainsi préparé, l’extrait est introduit dans une burette Mohr stérilisée, reliée, par un tube en caoutchouc, à une canule, et il est poussée dans una veine jugulaire externe par la force de la gravi-té, avec une vitesse moyenne de 100 cc pour 15 à 20 minutes (…).
L’attenuation du syndrôme diabétique commence immédiatement aprés l’injection (…).
Mais, avant l’injection, on prend, de la carotide, 24 cc de sang, pour doser la glycose, et 10 cc de sang pour doser l’urée.
De même, on reprend 25 cc et 10 cc de sang carotidien, pour doser la glycose et l’urée, immédiatement après l’injection, puis une heure plus tard, et ainsi de suite.
La séparation de la glycose du sang se fait par l’alcool à 96º. Son dosage dans le sang et dans l’urine, s’effectue par le procédé indiqué plus haut (T.I., p. 99).
La séparation de l’urée du sang se fait de la même façon que celle de la glycose. Son dosage s’effectue à l’aide de l’hypobromite de soude”.

English translation:

**Table:** 

Afterwards, taking meticulous aseptic precautions, this gland is chopped in a Latapie grinder, sterilised in the oven. Then, ten times its weight of sterilised distilled water is added to the preparation, and after shaking it several times, it is placed in the icebox.
After 24 hours, this diluted hash is filtered through a double sterilised tarlatan compress and 7 per 1,000 of NaCl is added to the filtrarum.
Thus prepared, the extract is introduced into a sterilised Mohr's burette, connected by a rubber tube to a cannula, and it is pushed into an external jugular vein by the force of gravity, with an average speed of 100 cc for 15 to 20 minutes (...).
The attenuation of the diabetic syndrome begins immediately after the injection (...).
But before the injection, 24 cc of blood are taken from the carotid artery to measure the glucose and 10 cc of blood to measure the urea.
Similarly, 25 cc and 10 cc of blood from the carotid artery are taken to measure glucose and urea immediately after the injection, and again one hour later, and so on.
Glycose is separated from the blood by using 96º alcohol. Its determination in blood and urine is carried out by the procedure indicated above (T.I., p. 99).
The separation of urea from blood is done in the same way as for glycose. Its determination is carried out with the help of sodium hypobromite.

Paulescu transferred the fresh gland under aseptic conditions to a Latapie mortar for its fragmentation, adding ten volumes of sterilized water, followed by agitation and refrigerated storage. Twenty-four hours later, the mixture was filtered through a double sterilized tartalan compress, adding a solution of sodium chloride (0,7%). Then the aqueous extract was administered to the pancreatectomized dog via a sterile Mohr burette, cannulated into a central or peripheral vein at a rate of 100 mL in 15–20 min. The attenuation of the diabetic symptoms appeared immediately after the intravenous injection.

### Pancreina

**Fig. 4:**
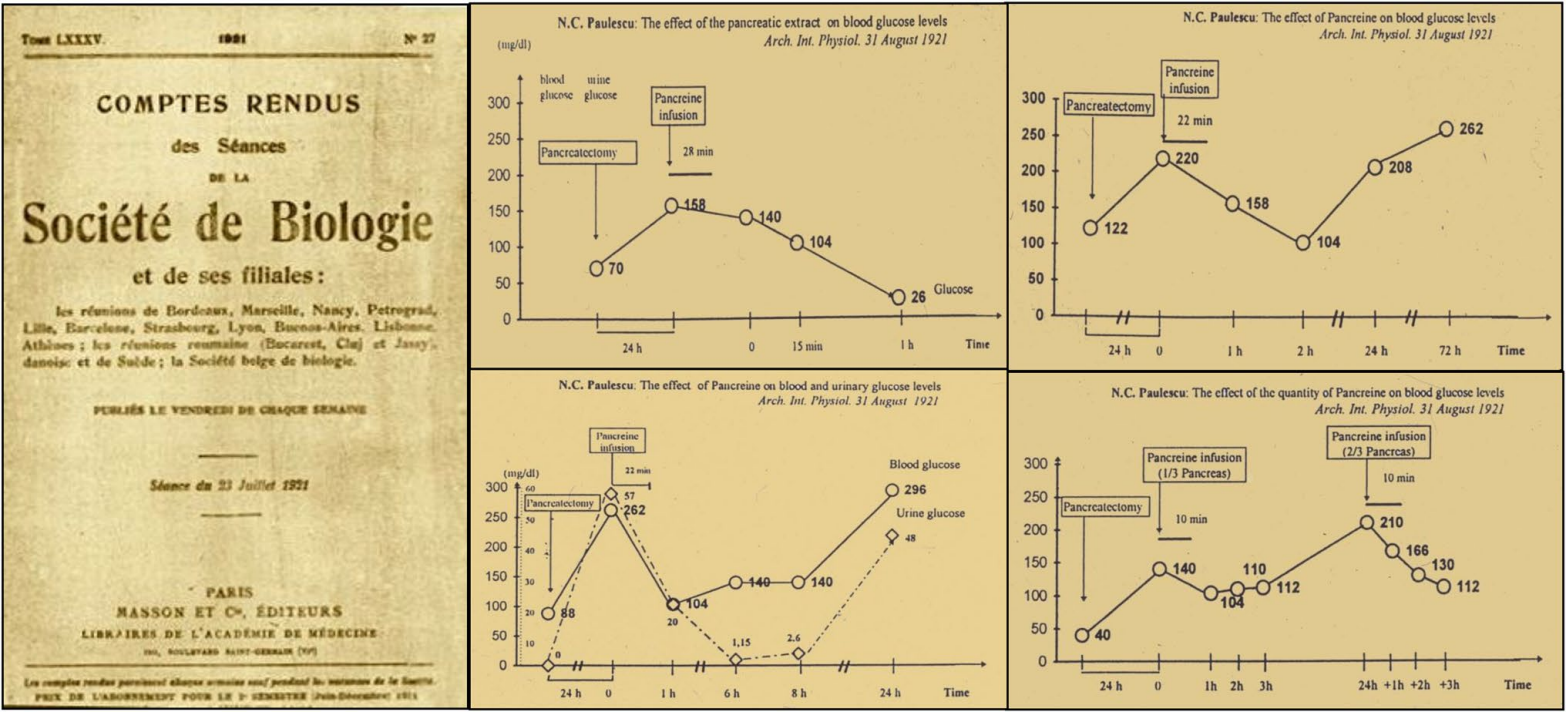
*Left* Nicolae Paulescu published four short articles covering nine experiments in CR Société de Biologie, showing the results of the administration of his pancreatic extract to dogs with experimental diabetes. *Right* The four graphs are from his long original article, published in August 1921. They illustrate: **a** the development of diabetes after complete pancreatic ablation; **b** the effect of parenteral administration of pancreina on the glycemic profile in the pancreatectomized dog; **c** serial study of the variations in glycemia and glucosuria over 24 h induced by the injection of pancreina; **d** evaluation of the intensity of the glycemic response in the animal with experimental diabetes depending on the amount of pancreatic extract administered [60]. (Courtesy of Constantin Ionescu-Tirgoviste)

Paulescu presented the results of the administration of his pancreatic extract in three scientific meetings of the Romanian Society of Biology (branch of the European Society of Biology) in Cluj (April 21), Iaşi (May 19) and Bucharest (June 23). As a consequence, four short articles covering nine experiments were published in the issue of *Comptes rendus des Séances de la Société de Biologie et de ses filials* (Paris)*,* dated on July 23, 1921. (Fig. 4). Main effects observed were:

1. The total ablation of the pancreas in the dog determined increased content of glucose, urea and ketone bodies in blood and urine. The injection of the pancreatic extract induced a temporary suppression of hyperglycemia, disappearance of glycosuria, and decreased concentration of urea and ketone bodies in blood and urine. Similar effects were observed when the extract was injected in a branch of the portal vein (mesenteric or splenic venula) or in the jugular vein [56].
2. The effect on blood sugar started soon after the injection, reached the maximum levels in about two hours and disappeared in approximately twelve hours. A parallel decrease of glucose excretion was associated to the reduction of the hyperglycemia [57].
3. The quantitative changes were relative to the amount of gland being administered. With a third of the pancreas, a slight reduction was observed; with two thirds, the effect was markedly intense [58]
4. In the non-depancreatized, non-diabetic dog with intact pancreas, the administration of the extract in the yugular vein generated transient hypoglycemia and reduction of urea levels in blood and urine [59].

**Fig. 5:**
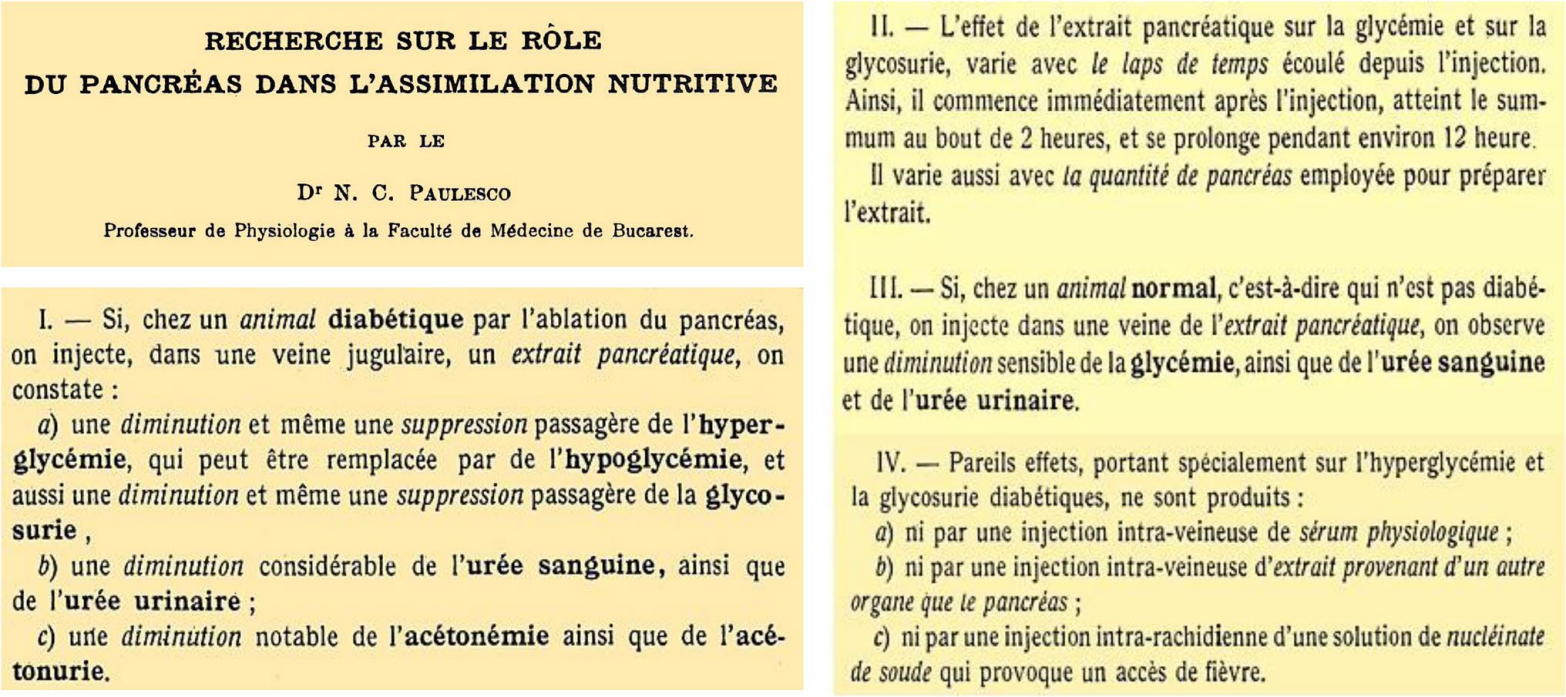
Main results and conclusions of the experiments carried out by NC Paulescu demonstrating the effects of pancreina on the metabolism of carbohydrates, proteins and ketone bodies and the absence of response observed in control experiments (other organs than the pancreas, saline and sodium nucleinate) [60]

On August 31, 1921, Paulescu published a large and comprehensive article, already accepted in June 1921 by *Archives Internationales de Physiologie* (Fig. 5) [60].

In the document, a summary of experiments performed along 20 years of research, demonstrated the hypoglycemic effect of the aqueous extract, in association to the reduction of glycosuria and ketonuria in pancreatectomized dogs. The manuscript displays the technique to achieve a sterile extract, the methods used to estimate blood glucose (procedure of Pflüger), glycosuria (as Claude Bernard), ketones (as Deniges), urea (sodium hypobromite), the clinical manifestations observed in the animals, informed autopsies, registry of body temperature, exact date and timing of each step and duration of each experiment. In the article he included new information focused in control experiments. The administration of normal saline and splenic extract did not reduce the hyperglycemia of pancreatectomized dogs. The administration of the pancreatic extract, that he named **pancreina,** generated fever as the main side effect. Paulescu induced a bout of fever (up to 38.9 °C) to the animals by the intraspinal injection of a solution of sodium nucleate; both hyperglycemia and glycosuria remained unchanged [37, 59, 60].

#### The patent of pancreina (1922)

**Fig. 6:**
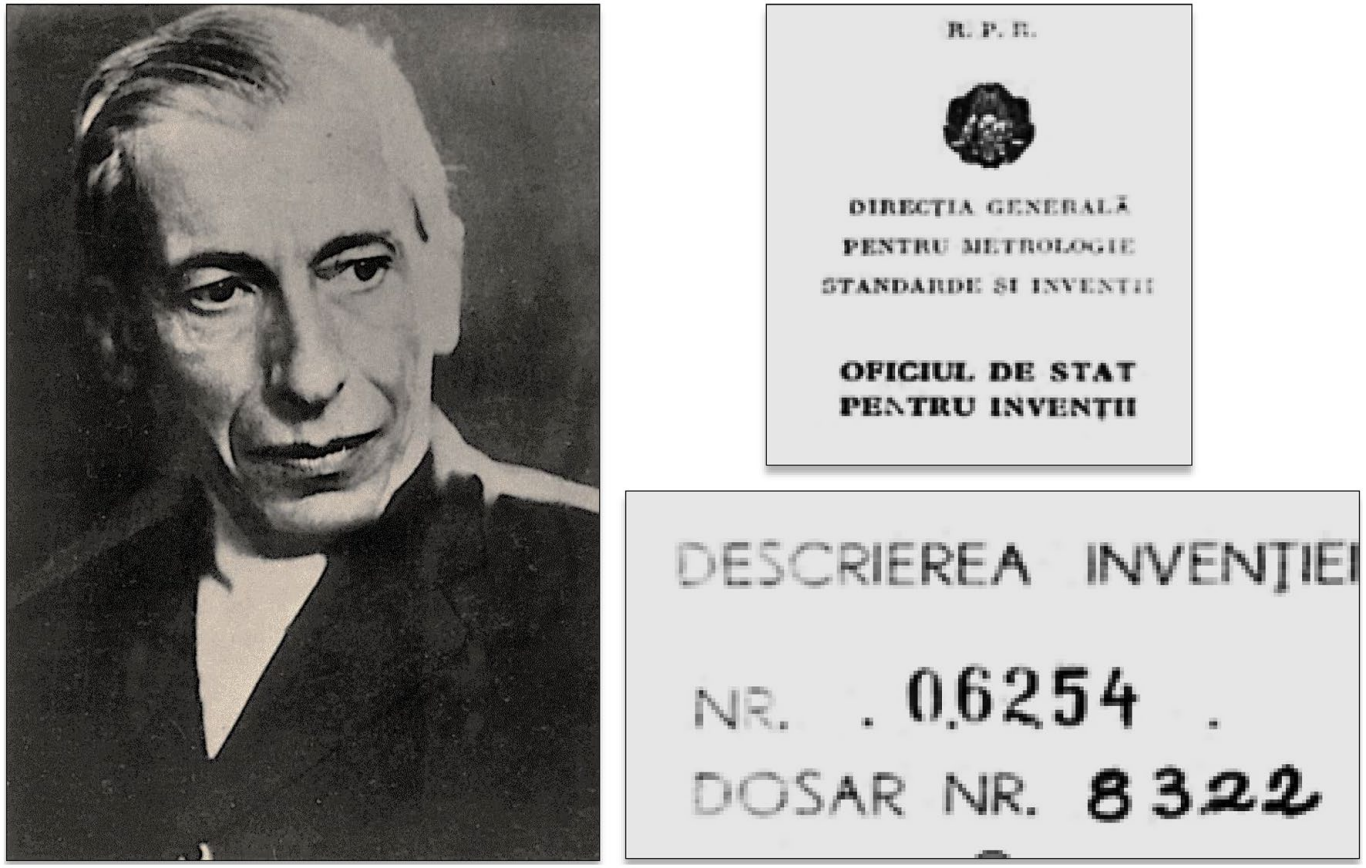
Nicolae Paulescu: Registration of the pancreina patent. Romanian Ministry of Industry (April 10, 1922)

On April 10, 1922, Paulescu successfully processed the registration to the Romanian Ministry of Industry of the patent of the pancreatic extract that he called pancreina (Fig. 6) [61, p. 83–84].

To purify pancreina, Paulescu subjected the aqueous glandular triturate to a double pad filtration of sterile hydrophilic gauze. Then, the addition of 1% hydrochloric acid generated a protein precipitate that was filtered again before neutralizing with caustic soda. The final stage of the process consisted of sterile filtration through a Berzelius leaf and evaporation by means of heat at a temperature not exceeding 50 °C [62].

Paulescu also carried out multiple experiments to optimize the administration of pancreina. To this end, he compared intravenous, subcutaneous, oral, and intrarectal routes, concluding that only parenteral administration (intravenous and subcutaneous) was effective. The limited clinical experience achieved with a scarce number of “thin” and “fat” diabetic subjects determined the interruption of the trial, due to the persistence of toxic effects, particularly, fever [37, 61, 63].

### Paulescu’s letters to Frederick Banting and to the President of the Nobel Commission in 1923

**Fig. 7:**
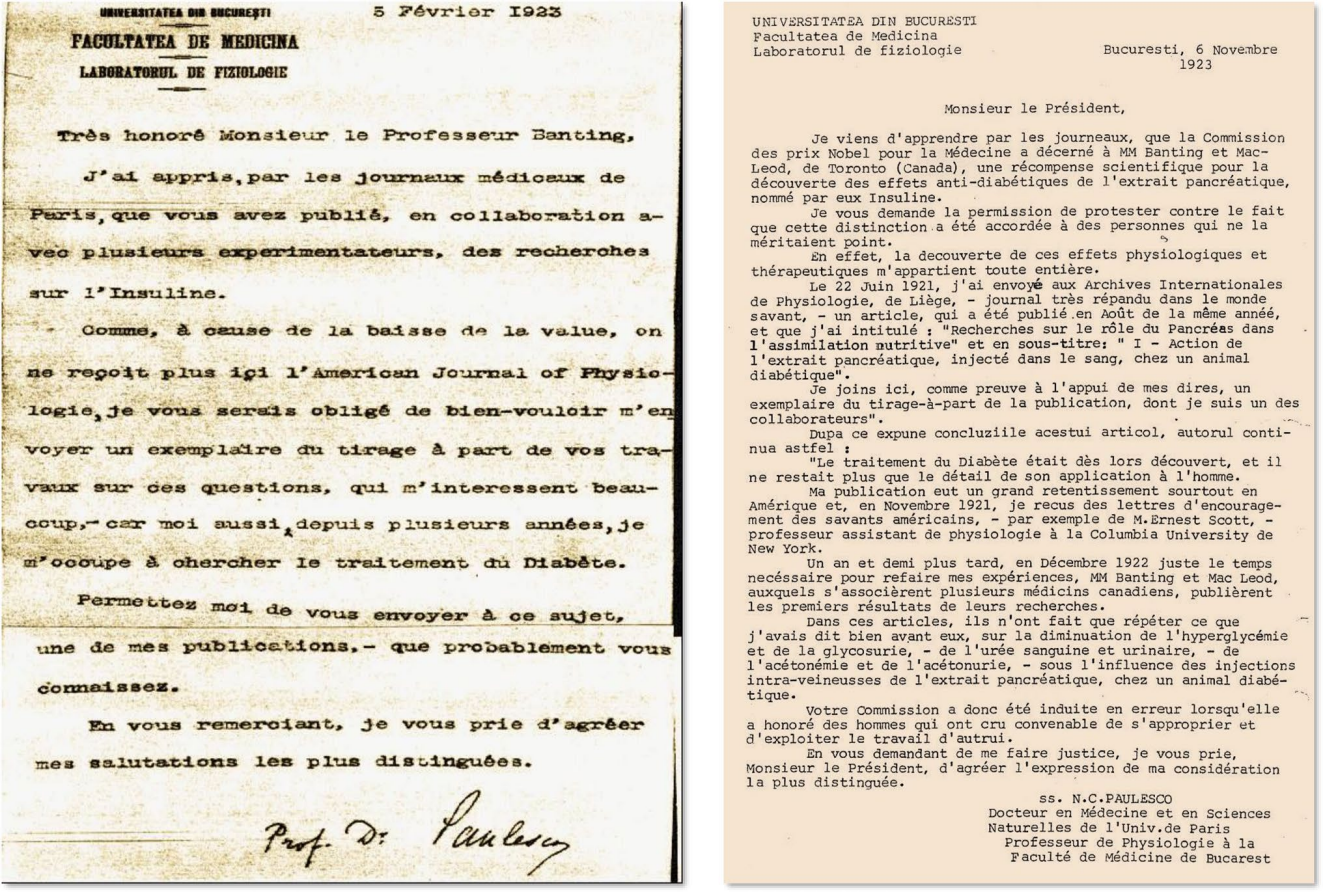
*Left* Letter from Nicolae Paulescu to Frederick Banting (February 5, 1923). University of Toronto Archive. *Right* Letter of protest from Nicolae Paulescu to the Chairman of the Nobel Commission for the award of the Nobel Prize in Physiology or Medicine to Frederick Banting and John Macleod (NC Paulescu personal papers; courtesy of Dan Angelescu)

On February 5, 1923, Paulescu wrote to Frederick Banting, enclosing his 1921 publications on the treatment of experimental diabetes with pancreina. He never got a response from the Canadian investigator. On November 6, 1923, Paulescu wrote to the President of the Nobel Commission, enclosing a copy of his publication in the August 1921 issue of *Archives Internationales de Physiologie*. He bitterly protested the Nobel award to Banting and Macleod and demanded justice, insisting on the priority of his publications on the discovery of the antidiabetic hormone and that the Toronto team had violated his intellectual property rights (Fig. 7).

**Table:** 

(…) Please, allow me to protest against the fact that this [Nobel] distinction has been awarded to people who do not deserve it. Indeed, the discovery of these physiological and therapeutic effects belongs entirely to me
(…) In these articles, they do nothing but repeat what I had already said, well in advance, about the decrease in hyperglycemia and glycosuria, blood and urinary urea, acetonemia and acetonuria, under the influence of intravenous injections of pancreatic extract, in a diabetic animal
NC Paulescu, November 6, 1923

The response of the Nobel Commission was to send the pamphlet entitled "The Nobel Prize Winners of 1923" with the award presentation speech by SJ Sjöquist, highlighting the contents of Banting and Best's first article of February 1922 [26].

In 1930. Paulescu wrote: “I previously believed that the scientist works safely because of the conviction that the date of publication protects him from any injustice. Unfortunately, today I am forced to admit that I was completely wrong” [64].

### International experts publicly expressed their solidarity with the vindication of Paulescu's scientific merits and recognized his priority in the discovery of the antidiabetic hormone (1923–1934)

The *Spitalul* medical journal published Paulescu's claim addressed to the Biological Society of Bucharest (affiliate of the Parisian), expanding on the chronology, results of his experiments and related publications between 1916 and 1921 [26].

In a letter dated November 5, 1921, Ernest L. Scott recognized the importance of Paulescu's findings, consistent with his experiments at Columbia University in 1921, which suggested the existence of a pancreatic hormone that regulates carbohydrate metabolism [26, 65].

John R. Murlin wrote in 1922 that results published by Paulescu in 1921 showed that intravenous administration of a sterile pancreatic extract to pancreatectomized dogs reduced and even temporarily eliminated hyperglycemia and excessive production of urea and ketone bodies [26, 32].

Alfredo Sordelli, President of the Academy of Exact, Physical and Natural Sciences of the Harvey Society, Academy of Sciences (New York) and of the Société de Biologie de Paris, acknowledged that Paulescu's results, published in 1921, were practically identical to those of the first publication, eight months later, by the Canadian researchers Banting and Best [66].

Casimir Funk, in addition to his extraordinary work in the field of vitamins, carried out extensive chemical studies on cancer and diabetes. In 1924, he wrote the following: “In 1920 and 1921, Dr. Paulescu of Romania decisively demonstrated that the pancreas contains an antidiabetic substance which has been given the name of insulin” [67].

Sir Edward Albert Sharpey-Schäfer briefly described the research activities of N.C. Paulescu [68]:

**Table:** 

(…) NC Paulescu experiments commenced in 1916 but interrupted by the German invasion [sic*] of Romania were published in 1921. They established that—in dogs rendered diabetic by extirpation of the pancreas and showing all the signs of severe diabetes-, (1) increase of sugar in the blood and its appearance in the urine; (2) increase of acetone bodies in the blood and their appearance in the urine; (3) increase urea in blood and urine—these signs can be made to disappear by intravenous injection of pancreatic extract. The extract he used was made from fresh pancreas with ice-cold distilled, “ stérile autant que possible”. It was introduced gradually into a vein of the diabetic dog, and its effect in a short time was to bring down the blood sugar level to normal or less, and to greatly reduce or abolish the glycosuria
(…) In later experiments attempts were made by Paulescu to isolate the active substance (termed by him pancreina) by submitting the aqueous extract to the action of acids and alkalis, of alcohol and heat. He found that boiling destroys the activity, and that an extract, from which most of the proteins have been precipitated by alcohol or by neutralizing the acidified extract by soda, contains the active substance. He further found that no effect was obtained by oral administration
E.A. Sharpey-Schäfer, 1926

[* Note: the invasion of Romania by the Austrian army, assisted by the German military forces].

In 1926, J.J.R. Macleod recognized Paulescu's scientific merits and his contribution to the discovery of pancreatic hormone, praising the experimental results published in the *Comptes Rendus de la Societé de Biologie* in April 1921, eleven months before the first article by Banting and Best [22; p.66]:

**Table:** 

(…) Paulescu’s researches were communicated at a meeting of the Reunion Roumaine de Biologie in the spring of 1921, when he described the effects produced by intravenous injections of sterile pancreatic extracts on the percentage of sugar, of acetone bodies, and of urea in the blood and urine of depancreatized dogs. There can be no doubt that all three substances became markedly reduced in amount, in blood and urine, as a result of the injections. The results were the same whether the injection was made into a branch of the portal vein or into a yugular vein
(…) The effects were noticeable in one hour following the injection, attain their maximum in two hours, and passed off in twelve hours. They varied with the amount of gland present in the injected extract
Paulescu also observed that the blood sugar, as well as the blood urea, in a normal dog became lowered by the injections
JJR Macleod, 1926

Wilfried Louis Batten Trotter (1872–1939), a prominent British surgeon and a relevant authority in the field of biosociology, spoke in the Hunterian Oratio Lecture (1932) some heartfelt words about Paulescu's contribution to the discovery of the antidiabetic hormone [26]:

**Table:** 

Paulescu’s research was the culmination of years of experimental work of precursors, colleagues and himself. This great advance, perhaps equivalent in some respect to the discovery of the therapeutic virtue of penicillin, remains unknowledged	
W.L. Trotter, Huntarian Oratio Lecture, 1932	

Paul Georg Trendelelenburg (1884–1931), Professor of Pharmacology at the University of Berlin, with research activity focused on endocrine pharmacology, praised Paulescu's scientific work in 1934 in the following terms: "Shortly before the discovery of insulin, Paulescu achieved complete success with pancreatic extracts that reduced blood sugar levels in pancreatectomized dogs within one hour of parenteral administration” [69].

### Paulescu died on July 7, 1931

Nicolae C. Paulescu died on July 7, 1931 from kidney complications related to a bladder carcinoma with a long and painful clinical course. He is buried in the family vault, Bucharest National Cemetery [26].

With the tragic ups and downs of the Second World War, the political problems in Romania and the rise to power of the Communist Party in 1947, the figure of Paulescu sank into oblivion. Paulescu, a radical antisemite, Orthodox Christian and member of the Romanian right, was considered an enemy of the regime. His traces were erased from the history of Romanian science [26].

## The discovery of insulin

### The first pancreatic extract (Banting and Best)

**Fig. 8:**
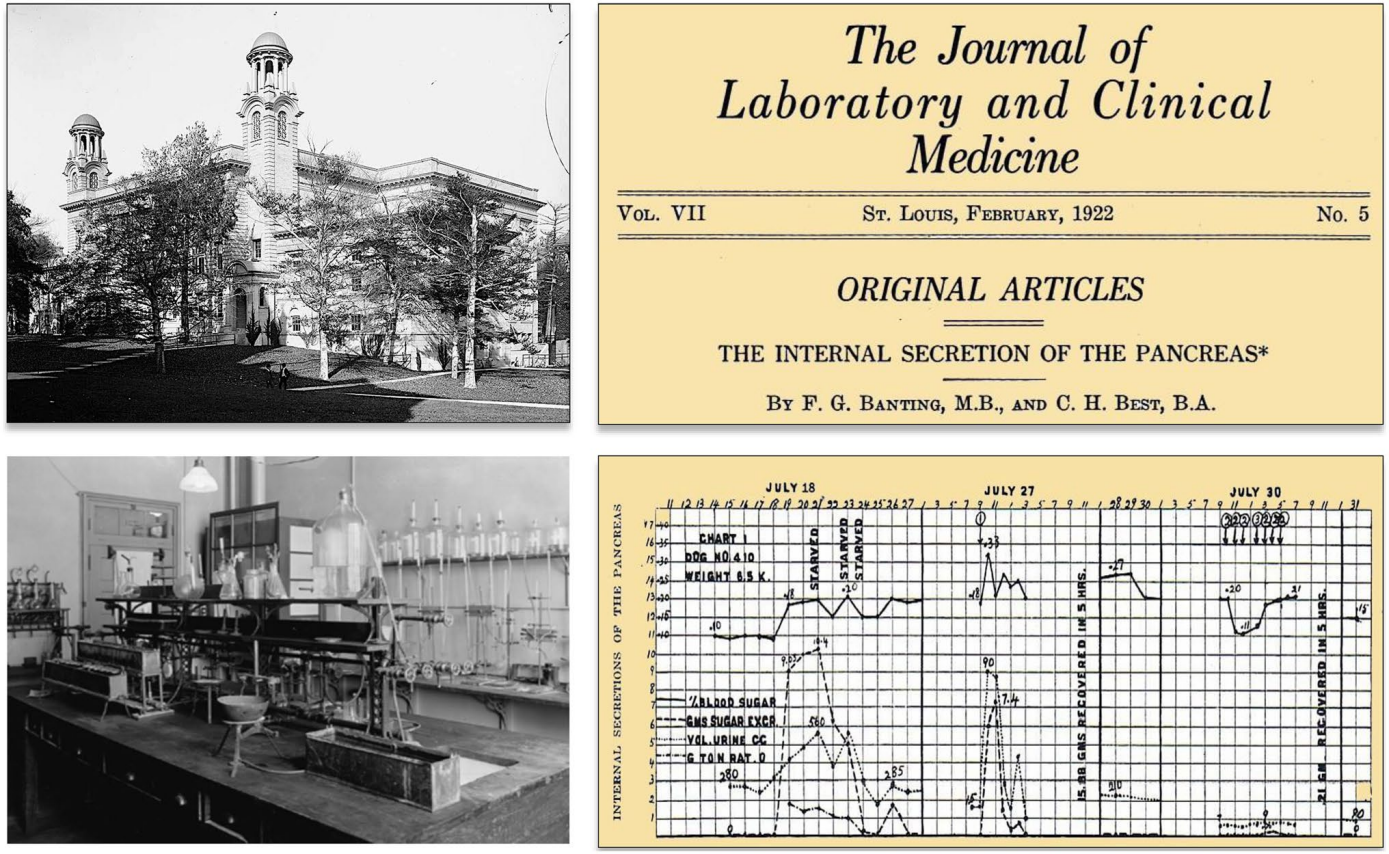
*Left* Photographs of the University of Toronto School of Medicine building and Laboratory of Physiology, University of Toronto; summer of 1921. Archives of the University of Toronto. https://nobelprize.orf/prizes/medicine/1923/banting/photo-gallery/. *Right* The first article of Frederick Banting and Charles Best (February, 1922) reported results of at least 75 doses of pancreatic extracts from “degenerated pancreas” [70]

On February 5, 1922, Frederick G. Banting and Charles H. Best published in the *Journal of Laboratory and Clinical Medicine* their first manuscript of the Diabetes Project, University of Toronto, describing the research activities performed by them from June to November 10, 1921 (Fig. 8) [70].

Banting and Best worked with the hypothesis that after a few weeks of having the pancreatic ducts tied off, the pancreas would atrophy but will contain the islands of Langerhans intact. Dogs were initially depancreatized by the method of Hédon (1909), pancreatic ducts were ligated under general anesthesia. A 10-week interval was considered necessary for complete degeneration of the acinous tissue. The removed pancreas was sliced into a chilled mortar containing Ringer’s solution. The mortar was placed in a freezing mixture and the contents partially frozen. The half-frozen gland was then completed macerated. The solution was filtered through paper and the filtrate, having been raised to body temperature, was ready for intravenous injection. Blood glucose estimations were made by the Myers-Bailey modification of the Lewis-Benedict method [71].

The article reported results after the administration of at least 75 doses of PE from “degenerated pancreas”. The main graphic of the manuscript showed that the administration of the “degenerated pancreatic extract”, developed by the two authors, only achieved a partial decrease of the levels of blood and urine glucose in just a fraction of the investigated pancreatectomized dogs. The authors felt that these results justified to state that this extract contained internal pancreatic secretion. The experiments revealed that the central idea of Banting, inspired to him by an article of M. Barron, “the ligature of the pancreatic ducts”, was not needed [72] (in fact, in experiments performed later they obtained better results using extracts from fresh adult and fetal pancreas). Barron’s hypothesis ignored that Heidenhain had demonstrated in 1875 that fresh pancreas extracts do not have proteolytic activity due to the existence of a zymogen able to generate an active enzyme only under various circumstances [73]. Bayliss and Starling confirmed Heidenhain’s observation, showing that the proteolytic enzyme trypsin was present in fresh animal pancreas as an inactive precursor (trypsinogen) [74]. Finally, Banting and Best decided on August 3, 1921 to replace the laborious Hedón’s procedure by one step complete pancreatectomy, discontinuing the ligation of the pancreatic ducts, acidulation of the alcoholic extract (suggested by Macleod), washing with toluol and use of the Berkfeld filter.

The first article of Banting and Best contained formal errors and meaningful mistakes as disagreements between the information supplied and figures or lack of accuracy in comments regarding the achieved results.

John James Rickard Macleod (1876–1935), director of the research project and well-known authority in the field (Professor of Physiology, director of the Department and President of the American Society of Physiology) decided not to sign the manuscript as coauthor.

The physiologist Ffrangon Roberts, University of Cambridge, published in the *British Medical Journal* a letter to the editor, before the end of the year 1922, expressing a severe critic of the experiments described by Banting and Best. He qualified them as “badly designed, badly conducted and badly interpreted” [75].

### Organization of diabetes treatment in the Department of Medicine at Toronto General Hospital, University of Toronto

Duncan Archibald Graham (1882–1974) was appointed in 1919 as the first director of the John Craig Eaton Chair of Clinical Medicine at the University of Toronto and Director of the Department of Medicine at Toronto General Hospital (TGH), positions he held until 1947. Graham hired Dr. Walter Ruggles Campbell (1891–1981) full-time, whom he assigned in collaboration with Dr. Andrew Almon Fletcher (1889–1964) to the Regular Admission Hospitalization Ward for patients with diabetes. These two expert clinics initially treated diabetes with a variant of the Allen's starvation diet [76].

Banting could not hide the anxiety that caused him the delay in starting clinical experiences with the PE. Prof. Duncan A. Graham didn’t authorize Banting to have direct contact with inpatients, considering that a surgeon, not on clinical practice, was not qualified for human experimentation. Nevertheless, the mediation by J.J.R. Macleod allowed for the administration of the first dose of PE prepared by Charles Best to Leonard Thompson, a 14-years old, charity patient with severe diabetes, first diagnosed in December 1919. On January 11, 1922 the resident physician Ed Jeffrey followed the order of the Senior Consultor Dr. Walter Campbell, to administer 15 mL of the Banting-Best preparation of PE (a turbid, light brown fluid), divided in two 7.5 mL injections, one in each buttock. Leonard had been admitted at the TGH on December 2, 1921. He was on the standard 450 kcal diet, which included 50 g of lean meat, vegetables and fruit, fat-free broth, clear tea and water, with a total carbohydrate provision of approximately 100 g. The patient was emaciated (weight, 29 kg) and had extreme glycosuria, severe ketonuria, hypotension, and a diuresis of 4 L in 24 h. The response to the administration of the PE consisted of light reduction in blood glucose from 440 to 320 mg/dL and in absolute glycosuria of 24 h from 92 to 84 g; ketonuria remained unchanged. A “sterile abscess” (characteristic of protein contaminants of the PE) rapidly developed in one of the injection areas. The extract failed to relief significantly the cardinal symptoms of the boy and its administration was discontinued [77].

### Collip’s extract

In December 1921, Macleod brought James Bertrand Collip to the team. Collip, a 29-years old biochemical expert, then Assistant Professor at the University of Alberta, was on sabbatical at UT with a Rockefeller Fellowship. Macleod asked him to help in the extract development. From the beginning, Collip worked with whole beef pancreas in his own laboratory.

On the evening of January 19, 1921, he was able to precipitate the active substance of the PE with ethanol at higher concentration than 90%. Using this threshold, he was able to remove most protein contaminants, obtaining a PE still with impurities but with a much higher potency than previously tested preparations [78].

**Table:** 

“To a small volume of 95 percent ethyl alcohol, freshly minced pancreas was added in equal amount. The mixture was allowed to stand for a few hours with occasional shaking. It was then strained through cheese-cloth and the liquid portion at once filtered. Some hours later, the precipitate was caught on a Buchner funnel, dissolved in distilled water, and then concentrated to the desire degree by use of the vacuum still. It was then passed through a Berkefeld filter, sterility test was made, and the final product was delivered to the clinic”
The precipitation of the active substance by 95% or absolute ethanol rendered the extract non-toxic. Essential points of the Collip’s extract were: minimal expression of protein, salt-free, lipoid free, and free of alcohol-soluble constituents

**Fig. 9:**
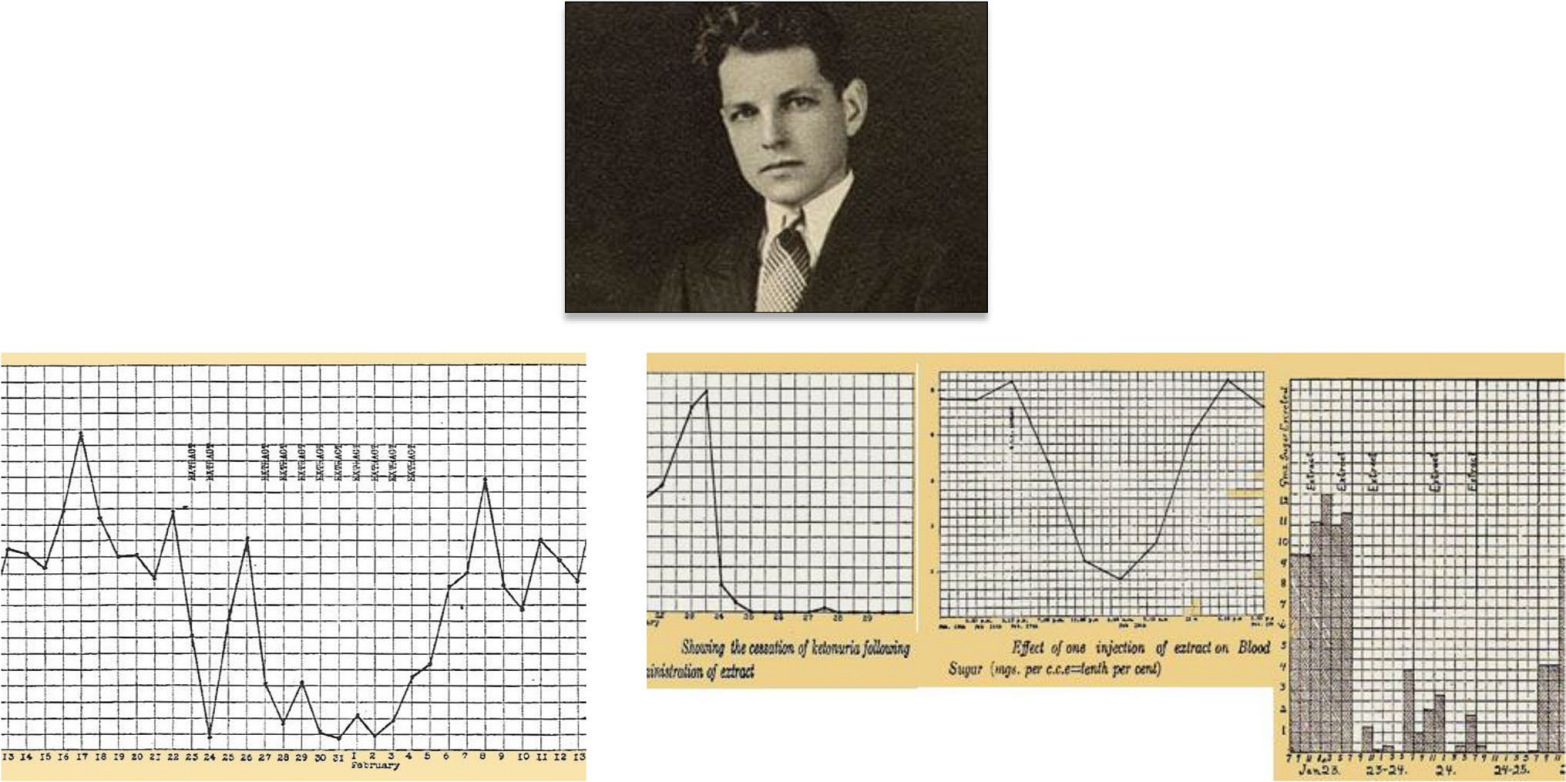
Portrait of Leonard Thompson (L.T.). Unknown date and author. University of Toronto Archives. Recorded effects of the administration of Collip's pancreatic extract to L.T., the first diabetic patient successfully treated in the Inpatient Ward of the Department of Medicine, Toronto General Hospital. *Left* Daily chart record between January 1 and February 20, 1922, of urinary glucose excretion. *Right* Rapid elimination of ketonuria. Reduction of blood glucose and glycosuria fractionated over 24 h. [79]

On January 23, 1922, Leonard Thompson was given the extract made by Collip. Blood glucose decreased from 520 to 120 mg/dL (Folin-Wu method), absolute glycosuria in 24 h from 71 to 9 g (Benedict method), and ketonuria (Van Slyke procedure) disappeared. The daily registry of absolute urinary glucose excretion during the months of January and February 1922 of L.T., demonstrated the superior effectiveness of Collip's EP compared to the previous of Banting and Best. In parallel to the excellent response in the amount of glucose excreted, a substantial reduction of blood glucose and urinary ketones was also recorded (Fig. 9), as well as clinical improvement. The successful results achieved with the case of Leonard Thompson were reproduced with six more patients during the month of February 1922 and published in March 1922 [79].

Prof. Duncan Graham assigned to Drs. WR Campbell and AA Fletcher the implementation of all features related with the trials with this new therapy.

### Announcement of the discovery of insulin on May 3, 1922

Between May 1921 and May 1922, research conducted by the team led by J.J.R. Macleod in the Department of Physiology, University of Toronto, demonstrated the actions of the PE on rabbit-induced hyperglycemia by various procedures (*piqûre,* subcutaneous injection of adrenaline, carbon monoxide, mechanical asphyxia), on glycogen stores in liver and other tissues, on the excretion of ketone bodies, on the respiratory quotient in healthy dogs with experimental diabetes investigated in metabolic cages) [80].

In experimental research, Collip used both normal and hyperglycemic rabbits. He could observe that blood glucose (BG) fell between approximately 25 and 50% within two hours after injection. For purposes of physiological assay, it was considered as most satisfactory to estimate the number of cubic centimeters which lowered the percentage of BG in the healthy rabbits to 0.045 in 2–4 h. The administration of 4 g of dextrose in 20% solution was followed by the recovery of the animals. These experiments were critical for the concept of unit of the dose of antidiabetic hormone [81–83].

The researchers of the University of Toronto decided to name **insulin** the hormonal preparation, ignoring that the name of *insuline* had already been proposed by the Belgian Jean De Meyer in 1909 [84], and E.A. Sharpey Schafer had proposed *insulin* in 1916 too [85].

**Fig. 10:**
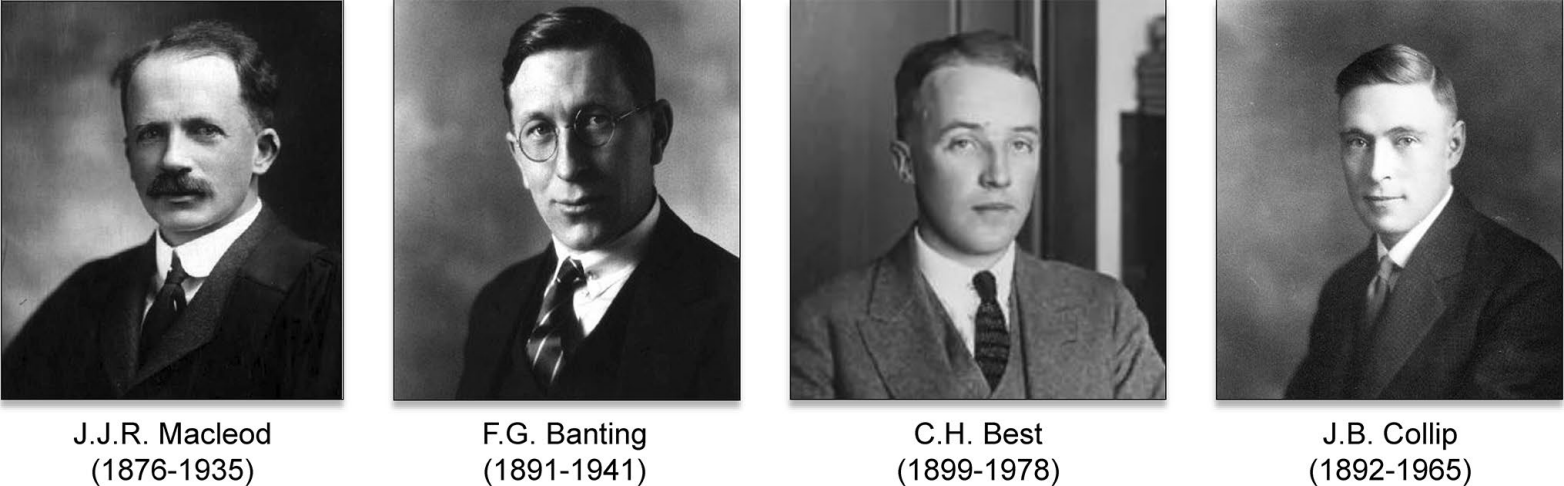
Between May 1921 and May 1922, the Diabetes Research Project led by Prof. JJR Macleod, Chair of the Department of Physiology at the University of Toronto, demonstrated the metabolic actions exerted by the pancreatic extract on healthy animals and the therapeutic effects observed in animals with hyperglycemia experimentally induced by various procedures. The photographs of the main authors of this research (JJR Macleod, FG Banting, CH Best and JB Collip) come from the Archives of the University of Toronto. Public domain

On May 3, 1922, at the annual meeting of the American Medical Association in Washington DC, Macleod summarized the research conducted by the departments of Physiology and Medicine at the University of Toronto, including animal experimentations and the clinical results achieved (Fig. 10).

At the end of the lecture, Macleod declared:

**Table:** 

These observations conclusively demonstrate that the pancreatic extracts that we have used contain substances that exert great potency on the control of carbohydrate and fat metabolism in both normal and diabetic animals, as well as in people with diabetes mellitus	

Given the impact created by this announcement, the date of May 3, 1922 has been considered, with scientific criteria, the date of the discovery of insulin.

### Collaboration between the University of Toronto (UT), Connaught Laboratories and Laboratories Eli Lilly & Co.

Between March and May 1922, attempts by Connaught Laboratories led by J.B. Collip to produce insulin on a large scale were unsuccessful. Diabetic patients in the spring of 1922 were in critical condition awaiting satisfactory insulin treatment.

An important progress in the purification of insulin had as its main protagonists Peter Joseph Moloney and Donald Munro Findlay who, in order to deal with the decrease in potency of the extract, used as an alternative the precipitation with benzoic acid of the active product of the pancreatic extract, followed by the treatment of the precipitate with ether and water, separating the two layers formed (the benzoic acid was contained in the ether layer and the insulin in the water layer) [86].

The aqueous solution was subjected to prolonged heating in a vacuum chamber for complete removal of the ether. The use of the Moloney and Findlay method was of critical importance for the availability of insulin in the most difficult stage of the Connaught and University of Toronto laboratories. More than 250,000 units of insulin made by this method were used in the treatment of diabetes patients with satisfactory results [87].

Collip was unable to solve the problem of the loss of potency of the pancreatic extract, associated with the need to increase its production on an industrial scale. Macleod and Sir Robert Falconer, President of UT, agreed to negotiate with Eli Lilly & Co. and its director of research, George Clowes, in the face of the urgency of manufacturing insulin in large quantities.

To do this, they created the Insulin Committee-University of Toronto (IC-UT), made up by members of the UT Governing Board, including its Chairman, Colonel A.E. Gooderham and Sir Robert Falconer, among others, an Advisory Committee that included the director of Connaught Laboratories, Dr. J.G. Fitzgerald, project investigators and members of the University, Drs. J.J.R. Macleod (Secretary), F.G. Banting and C.H. Best, and on behalf of Toronto General Hospital, Prof. D.A. Graham. The Committee had the legal advice of Mr. C.H. Riches [88].

The agreement was signed on May 31, 1922. According to the agreement, Lilly had "an experimental year" for the development of the indicators established by the UT and during this period the company would enjoy the exclusive rights for the production, use, sale and distribution of the PE in the USA, Mexico, Cuba, Central America and Latin America. Lilly would provide UT with 28% of each approved lot of insulin for free distribution, and 12% at cost, and batches of the pancreatic extract would be distributed as “tests” at production cost or free, to physicians and institutions selected by joint decision, After the conclusion of the experimental phase, Lilly would enjoy the license right on the condition of payment of 5% for the real rights of sales to UT.

This document represented the first agreement of cooperation between an academic institution, medical professionals and a commercial company with the aim of applying a therapeutic advance of great international relevance. Clowes appointed George B. Walden as supervisor of insulin production, under whose direction Harley W. Rhodehamel and Jasper P. Scott collaborated on the large-scale experimentation and processing projects [89].

Among the first modifications introduced by Lilly we must mention the use of frozen pancreas, the addition of acid in the extraction process and the isoelectric precipitation of insulin. In the month of October, Lilly was supplying the needs raised by 16 Canadian and North American clinical teams. The process of isoelectric precipitation of insulin ended in December 1922. In the winter of 1923, Eli Lilly finally obtained a more pure and stable insulin, less allergenic, in sufficient quantity, between 80,000 and 90,000 units per week, to meet world needs [90, 91].

### JJR Macleod definitely established that islet tissue is the source of insulin

There was as yet no absolute proof that the hormone of the pancreas was elaborated by the islet cells. In the summer of 1922, Macleod conducted experiments comparing the metabolic actions of pancreatic extract from elasmobranch and teleost fishes administered to rabbits. He gave recognition to the previous observation of Rennie that the islet tissue in the bony fish (Teleostei) is enclosed into nodules, frequently encapsulated and separated from the acinar tissue. J. Rennie and T. Fraser had failed to demonstrate the presence of the antidiabetic hormone because they preferentially administered the islets by mouth [92].

**Fig. 11:**
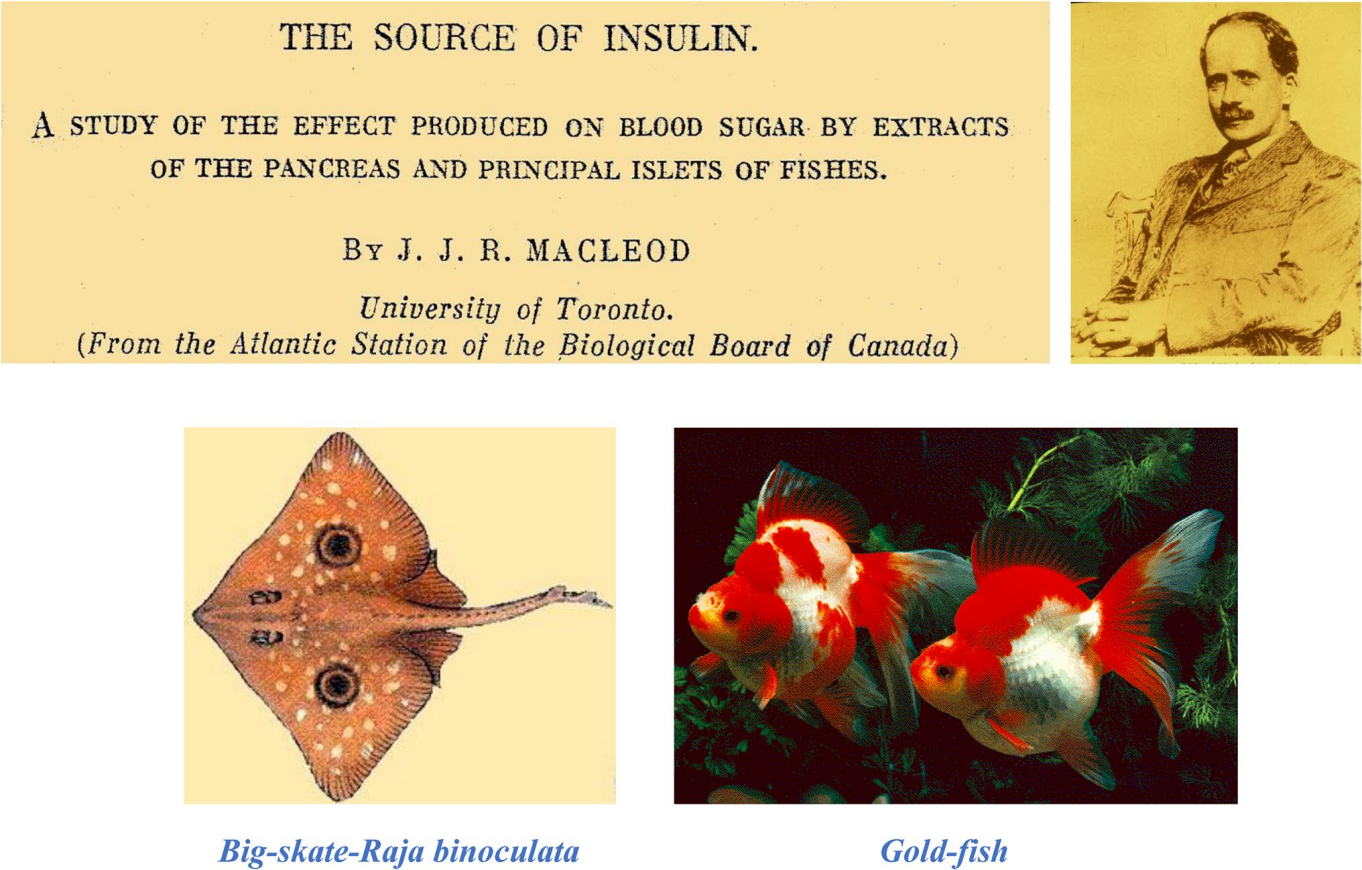
In the summer of 1922, JJR Macleod investigated alone at the Marine Biological Station in St. Andrew's, New Brunswick, the comparative effect of administration of islet-free acinar pancreas from elasmobranch fishes with administration of islets of Langerhans encapsulated in nodules and separated from the acinar tissue of bony fishes (teleostei). Macleod also performed control experiments with liver extracts [93]

The comparison made by Macleod between the administration of the islets and the pancreatic zymogenous tissue (containing no islets) leaved little doubt that insulin was present only in the islet tissue and not in the acinar tissue. He determined in different times the level of blood glucose in normal rabbits injected with extracts prepared from 1-the pancreas of representative Elasmobranchs (Squalus -dog fish-, and Raja -skate-); 2-Myoxycephalus -sculpi-, and Lophius piscatorius -angler fish-); c-the zymogenous (acinar) pancreatic tissue, as free as possible from islets, in the same and other Teleostei. Macleod obtained, at least, 3–4 units of insulin from one teleostei fish. Control experiments administering liver extracts showed no endocrine effects (Fig. 11) [93].

### Insulin: Canadian and US patent offices

On November 10, 1922, the University of Toronto applied to the USA office for the first patent for insulin with assignment to Best and Collip as investigators. The request was rejected on the grounds that it contravened the protection rights corresponding to the patent for acomatol granted on May 28, 1912 to Georg Zülzer. New applications were processed showing the benefits of the new PE in comparison to the PE of Zülzer: a—possibility of continuous administration; b—lack of serious adverse effects; c—greater potency and purity, and d—evidence of benefits for the treatment of diabetic patients [94].

**Fig. 12:**
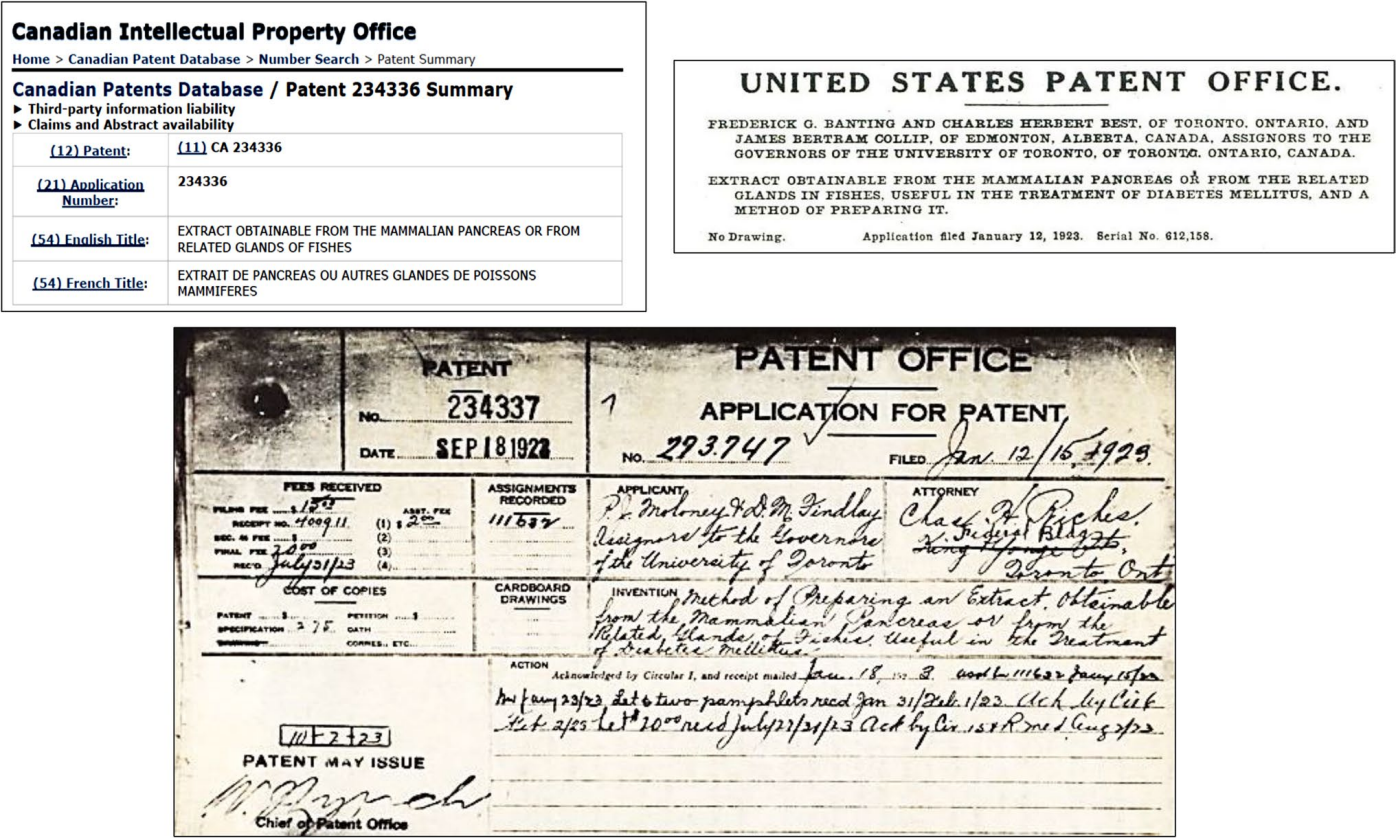
Insulin Patents. Patents 234,336 were issued to inventors FG Banting and CH Best and 234,337 to inventors PJ Moloney and D.M. Findlay (Canadian Intellectual Property Office) on September 18, 1923. Patent 1,469,994 (United States Patent Office) was granted on October 9, 1923. All of them were assigned ownership to the University of Toronto Board of Governors. In the absence of a fully purified substance and its clinical formulation, the patented product (extract obtainable from the pancreas of mammals or fish) was defined by a sufficient degree of purity that surpassed that of the preceding preparations, as well as its physiological effects and beneficial therapeutics for patients with diabetes [94]

Patents #234,336 (Canadian Intellectual Property Office; inventors: Banting and Best) and #234,337 (Canadian Intellectual Property Office: inventors P.J. Moloney and D.M. Findlay) were granted on September 18, 1923, and patent #1,469,994 (United States Patent Office; inventors: F.G. Banting, C.H. Best and J.B. Collip) dated October 9, 1923. All patents were assigned in ownership to the Board of Governors of the University of Toronto (Fig. 12).

In the months of May–June of 1923, the relations between Eli Lilly and the UT experienced serious conflicts which represented a danger for the continuation of the established collaboration. Clowes sent several letters to Macleod asking to accept Lilly's decision to file a new patent application on its own based on the development of a new extraction procedure. Clowes also told him that the company wanted to extend the experimental period and license the patent exclusively, claiming the intention to manufacture insulin at a lower cost, sell it at a lower price and facilitate free access to indigent patients. Macleod replied that the UT flatly rejected the proposal and added that the Insulin Committee-UT (IC-UT) had authorized the British Medical Research Council (MRC) to supervise the production of insulin in England [95].

Despite all of the above, on September 1923, George B. Walden worked up an American patent for "a new process for the preparation of pancreatic extract" without prior notice to the IC-UT. The procedure showed that the amount of available precipitated hormone depended on the concentration of hydrogen ions in the medium and that the best results had been observed with a pH between 4.5 and 5.5. In addition, it reduced the residual nitrogen content to 0.1 mg per unit of antidiabetic activity and the purity of the preparation was 10 to 100-fold higher than the best previously used pancreatic extract. The process, developed by Lilly, made the authorized procedure for patents granted to UT obsolete. With the granting of this patent, Lilly's monopoly for the production and sale of insulin in the countries of the American continent was foreshadowed [96].

George Riches, legal representative at the IC-UT, wrote a letter to Macleod on April 3, 1923, informing him of his concern about the risks posed by the granting of the Walden patent to the Eli Lilly firm against the interests of the University of Toronto and the validity of the Banting, Best and Collip insulin patent [97]. The IC-UT addressed the problem through two initiatives. The first was to inform the Council on Pharmacy and Chemistry of the American Medical Association that it did not accept the use of Iletin by the Lilly firm. The second was to contact P.A. Shaffer, director of the Biological Chemistry Laboratory at Washington University in St. Louis with the proposal to apply for a new patent. The research team led by Shaffer had independently published in 1923 the development of a new pancreatic extract using a process similar to that of Walden [98].

**Fig. 13:**
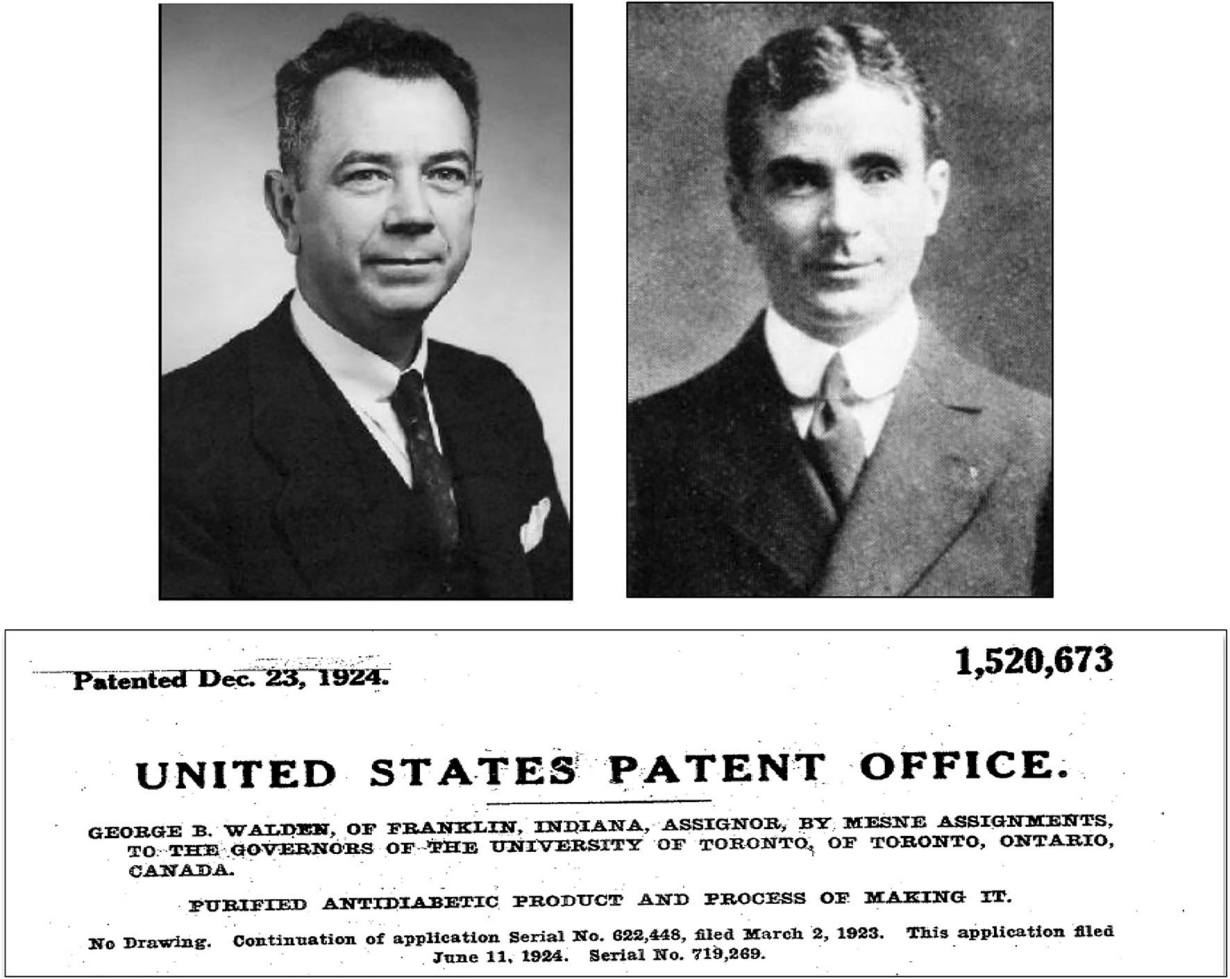
In the summer of 1923, a conflictive situation arose between Eli Lilly and the University of Toronto due to the intention of G. Clowes, Director of Research at Lilly, to apply for a new patent based on the development of a new exclusive extraction procedure, invented by G.B. Walden, showing that isoelectric precipitation markedly reduced the residual nitrogen in the preparation and increased the purity of the preparation between 10 and 100 times compared to the standard product. The pressure exerted by the Insulin Committee of the University of Toronto finally decided to assign the intellectual property shared with the University of Toronto: US Patent 1,520,673. Inventor George B. Walden. Owners: The Governors of the University of Toronto (December 23, 1924). Photographs of G.B. Walden and G. Clowes are from the Lilly Archives [99]

Given the pressure exerted by the IC-UT, Lilly finally decided to assign the intellectual property of the Walden patent to the UT and keep the name of its Iletin brand (Fig. 13) [99].

**Table:** 

“While we consider ourselves legally and morally entitled under our agreement with the committee to take the strongest possible patents on our own discoveries and whilst we are not in the least concerned about Shaffer’s claims as our process is superior to and differs essentially from his and we are satisfied of our priority, nevertheless, we would not consider doing anything that may embarrass Toronto University.”
(Clowes, 1923. Telegram to Professor Macleod, April 8, 1923)

Both parties agreed to extend the Walden patent licenses to foreign countries, considering it more effective than the one initially granted to Banting, Best and Collip [100].

### Unit of Insulin: standards (1922–1923)

The University of Toronto Insulin Committee organized on November 25, 1922 a round table in the library of the Faculty of Medicine with the participation of Canadian and American experts in the treatment of diabetes to discuss the organization of clinical trials and the insulin unit standardization plan (Frederick M. Allen, Rockefeller Institute of Medical Research, New Jersey; H. Rawle Geyelin, Columbia University Presbyterian Hospital, New York; Elliot P. Joslin, Harvard University, Boston; Russell M. Wilder, Mayo Clinic, Rochester, Minnesota; JR Williams, Rochester, New York; Roland T. Woodyatt, University of Chicago; William D. Sansum, Potter Metabolic Clinic, Santa Barbara, California, among others).

The discovery by J.B. Collip of insulin-induced hypoglycemic seizures in the rabbit allowed an initial approach to measuring the dosage of the new hormone. The UT researchers proposed considering the unit of insulin as the amount of extract (in cc) capable of causing a 45% decrease in the normal value of blood glucose in a healthy 2 kg rabbit (subcutaneous administration).

The IC-UT decided on December 30, 1922 to adopt a criterion for the "clinical unit of insulin" and another for the "physiological unit of insulin" [101].

**Table:** 

“The unit adopted for insulin shall be approximately one-fifth that of the original Toronto unit, which is the amount of insulin required to lower the blood sugar of a 2 kg. rabbit to 0.045% within four hours, and caused symptoms
The small unit would be more practical in cases were small amounts of insulin would have to be prescribed. By using the original (Toronto) unit, fractions would have to be prescribed, and this would be undesirable.”
(IC-UT, December 1922)

The Standards Commission, created by the Health Organization of the League of Nations met in Edinburgh in July 1923. It approved the IC-UT definition of the "physiological unit of insulin", and agreed that the "clinical unit of insulin" corresponded to one third of the value of the physiological unit of insulin [26].

### Insulin treatment in the months which followed its discovery (short report)

A special issue of the *Journal of Metabolic Research,* published a series of manuscripts covering the main experimental and clinical contributions dealing with insulin research in the months following its discovery.

A detailed presentation of nine insulin diabetic subjects treated at TGH was followed by highly relevant observations of W.R. Campbell about the first fourteen cases of diabetic coma attended [102, 103].

Campbell provided an outstanding lesson about the treatment of ketoacidosis, which, in many aspects remain valid today. He pointed out the extraordinary tolerance to insulin in coma and the key role of water, insulin, glucose and alkali in the treatment. Pros and cons of the administration of bicarbonate were discussed [104].

The third manuscript, signed by A.A. Fletcher and W.R. Campbell described the adrenergic and neurogenic stages of insulin-induced hypoglycemia. They observed severe reactions with a blood sugar of 60 mg/dL. A blood sugar value of 35 mg/dL was associated to unconsciousness. Different treatments of hypoglycemia were tested: orange juice (when the patient could swallow), intravenous glucose (5–20 g), epinephrine (1/1000 sol., 1 mL) when the patient was unconscious [105].

A recent publication associates clinical reports, incidents and biographical anecdotes related to the introduction of insulin therapy in North America and the European continent [106].

Walter R. Campbell and Andrew A. Fletcher of the TGH Department of Medicine (directed by Duncan A. Graham) successfully treated more than fifty patients with insulin before the end of 1922 at the Diabetes Ward of TGH [107].

By early 1923, more than 1000 diabetic patients were treated with *insulin or iletin* in 60 clinics of United States and Canada [26].

### The disclosing of documents explaining the true story of the discovery of insulin

A group of friends and admirers of Banting decided, from the first months of 1923, to orchestrate a campaign aimed at providing maximum credit for the discovery of insulin to F.G. Banting, Canadian hero of the First World War, wounded at Cambrai, relegating the Scottish emigrant J.J.R. Macleod, shy and aloof physiologist.

Banting's friend and colleague, Dr, George W. "Billy" Ross, son of Sir William Ross, who had been Premier of Ontario, with strong political connections, led the initiative. Ross was aided by Velyien Henderson, Professor of Pharmacology, UT, Sir William Mulock, Vice-Chairman of the UT, and Charles Evans Hughes, previous Republican candidate for the USA presidency against Woodrow Wilson, Secretary of State in the administration of USA President Warren Harding (1921–1925), and later appointed president to the Supreme Court between 1930 and 1941. At all times, Fred Banting had the support and favors of William Lyon Mackenzie King, the Prime Minister of Canada.

With Banting's consent, Ross wrote to all known Canadian expert diabetologists asking for their support to Banting and the miracle of the discovery of insulin. As a consequence of this campaign, the Canadian Medical Association passed a resolution on March 23, 1923 (also approved by the Toronto Academy of Medicine) dictating that the internal secretion of the pancreas, named insulin, was discovered by Banting and Best in the summer of the year 1921 [108].

The UT decided to create the Banting and Best Chair of Medical Research, a special professorship to Banting with an annual grant of 10,000 CAD to pay for his salary and research support, plus an additional amount of CAD 10,000 as reimbursement for the discovery period. Also, on June 27, 1923 the Canadian House of Commons accepted a resolution to grant Banting with a lifetime annuity of CAD 7,500 to devote his life to medical research. On the contrary, and particularly in Canada, the role in the discovery of insulin of the Scottish introverted immigrant, Macleod, physiologist and not clinician, was forgotten [109, pp. 214–222].

The animosity and confrontation of Banting and his admirers against Macleod persisted for a long time. Finally, Macleod accepted the nomination of Regius Chair of Physiology at the University of Aberdeen, his *alma mater*, and left Toronto in 1928. One friend reproduced the last words of Macleod before departure: “I am shuffling my feet on the floor to wipe away the dirt of this city”. At the University of Aberdeen, Macleod carried out a notable teaching activity and resumed his research on the influence of the nervous system on hepatic glycogenolysis. In 1935 he updated the edition of his book *Physiology and Biochemistry in Modern Medicine*. Progressively debilitated by rheumatoid arthritis, he died in 1935 at the age of 59 years with symptoms of cardiorespiratory failure [109, p. 234]. His passing temporarily coincided with the sudden death of another professor at the university. A joint funeral was held in King's College Chapel. Macleod's remains rest with those of his wife, Mary Watson, in Aberdeen's Allenvale Cemetery. Macleod was relegated to oblivion, as was his leadership in the discovery of insulin.

### Banting, responsible for and organiser of the Anglo-Canadian germ warfare programme (1938–1941)

After the discovery of insulin, Banting supervised research on silicosis developed by scientists of the Banting Research Department but never had real success in his own research initiatives.

On 11 September 11, 1937, Banting warned General Andrew McNaughton, Chairman of the National Research Council, that Germany was prepared to initiate biological warfare (which never could be demonstrated). In 1938 he was elected the first Chairman of the Associate Committee on Medical Research of the National Research Council and rejoined the Royal Canadian Army Medical Corps with the rank of major. With McNaughton’s encouragement, he prepared a detailed memorandum of biological warfare, describing the various air-borne, water-borne and insect-borne agents which could be used for military purposes. His confidential program (paid with 1.3 million CAD) to eliminate the German enemy consisted of creating epidemics, famine and economic misery through the attack on livestock, sterilization of crops, food poisoning and contamination of water and fields.

In September, 1939, Banting entered the Canadian Army with the rank of major. In November, he updated the germ warfare program memorandum and handed it over to Lord Maurice Hankey, the British Minister, coordinator of the subcommittee on germ warfare. McNaughton informed Banting of the final agreement between Great Britain and Canada on the joint germ warfare program and the decision to appoint him as delegate of the Canadian government for all purposes.

Banting created a core group of Canadian biological war researchers including the bacteriologists P.H. Greey and D. Fraser of UT and the virologist James Craigie of the Connaught Medical Research Laboratories, among others. C.J. Mackenzie provided Banting with CAD 50,000 of seed money out of the so-called Santa Claus Fund until British support was forthcoming [110, pp. 14–55].

The British military high command decided to install the bacteriological warfare arsenal in Porton Down (England), in accordance with the instructions provided by Banting. The first proposed offensive action on German territory would consist of the aerial drop of sawdust infected with lethal infectious agents. The preparatory tests were made at Balsam Lake, some 50 miles northeast of Toronto, between October 9 and 16, 1940 [111, pp. 1–18].

After these tests were completed, Banting noted in his personal diary: “We are beyond the purely experimental stage (…) for obtaining the means by which we can retaliate 100-fold if the Germans used bacterial warfare (…) We have to kill 3 or 4 million young huns—without mercy—without feeling” (October 14–20, 1940) [112, pp. 283–286].

The “Billy” Ross documents reveal the deteriorating relationship between Banting and Best. Banting disparaged his former aide on several occasions. Charles Best wanted a Research Institute to be established and named after him. Banting immediately set about frustrating this plan. “Best is naïve in his abject selfishness”, he said. In 1940, Best offered himself as the Canadian’s government medical emissary to a besieged Britain. Banting was fully aware of the dangers involved in the crossing, he elected to go himself. Shortly before leaving, he was heard to say: “If they ever give that chair of mine to that son of the bitch, Best, I will roll over in my grave”. This was reported by Australian John Waller, Ph.D. in History from University College London and Research Fellow at the Wellcome Trust Center for the History of Medicine, in his book entitled *Fabulous Science* [113, p. 232].

The Anglo-Canadian military steering committee called a meeting in London to decide on joint military actions against the enemy. Banting's secret mission was deemed "of high scientific and national importance" by the Canadian Prime Minister.

**Fig. 14:**
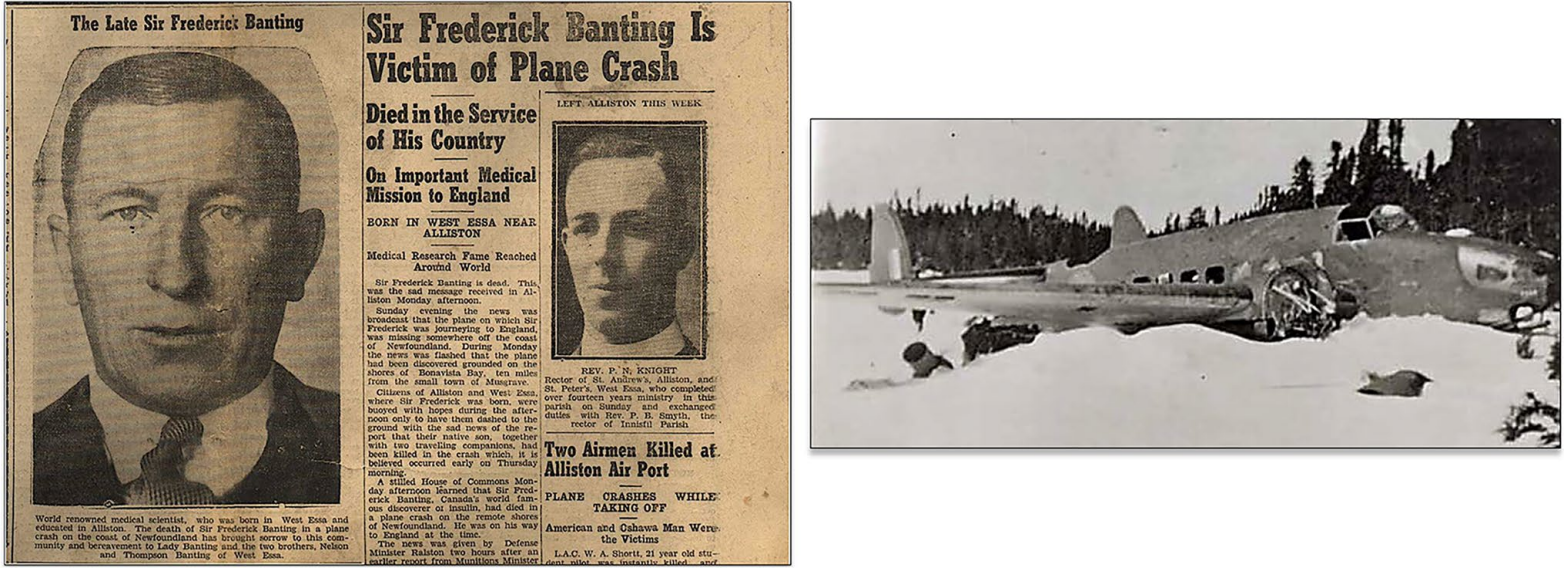
Sir Frederick Banting, victim of plane crush (February 20, 1941). News report on the accident in the Alliston Herald. Public domain

At around 8 pm on February 20, 1941, a few minutes after taking off from the Gander military airport, the bomber had mechanical problems. The first pilot, Joseph C. Mackey, attempted an emergency landing and crashed in the grove near Musgrave, Newfoundland. Both the radio specialist and the second pilot died immediately. Banting was mortally wounded and died in the snow the next day. Only Mackey survived (Fig. 14). The body of Banting was flown to Toronto. On March 4, the solemn funeral was held in the UT Convocation Hall. The casket was carried in solemn procession to Mount Pleasant Cemetery where Banting's remains rest alongside those of his second wife Henrietta Ball [112, pp. 298–310].

### Charles Best reinvented the history of the discovery of insulin (1941–1978)

After Banting's death, the UT presidency decided that Best should succeed him as director of the department. Best considered that the occasion had come to spread a new reading of the discovery of insulin, attributing to himself the exclusive role of the preparation and administration of the pancreatic extract to the first patient.

In his account he backdated the great discovery to the first weeks of the summer of 1921, when Banting and he worked alone [113, pp. 238–239].

**Fig. 15:**
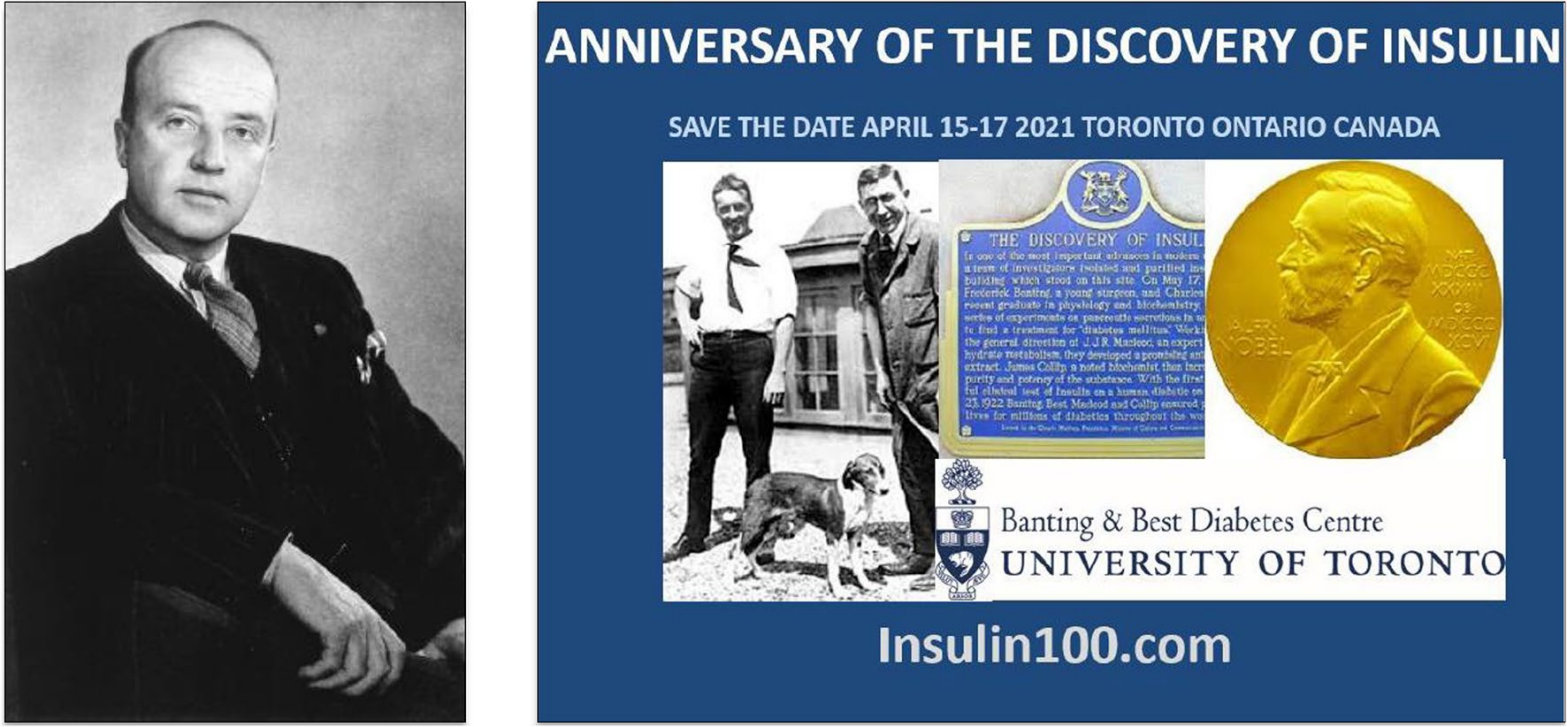
Charles Best backdated the discovery of insulin to the first weeks of the summer of 1921. The Banting and Best Center of the University of Toronto celebrated the centenary of the discovery of insulin—insulin100.com -, on April 15–17, 2021, advertised with only images of researchers Banting and Best

His maneuver prospered to the point that most organizations celebrate the anniversary of the discovery of insulin in 1921 and not in 1922. As an example, the University of Toronto celebrated the 100th anniversary of the discovery of insulin (insulin100.com) on April 15–17, 2021, that was advertised with images of Banting and Best, excluding Macleod and Collip (Fig. 15).

Best knew how to position himself at the forefront of the most important national and international organizations in diabetology. For this purpose, he had the favor of academics of maximum influence. Among them, Prof. Henry Dale, Nobel Prize in Medicine in 1936, and Dr. Robert Daniel Lawrence, diagnosed with DM in 1920. Lawrence and the writer George Wells (also suffering from diabetes), created the British Diabetic Association in 1934. Dale, Lawrence, and Best were co-founders of the American Diabetes Association and the International Diabetes Federation.

Best convinced the UT authorities to keep secret for 56 years Macleod’s personal account, written in September 1922, about the events and activities carried out in the Department of Physiology since May 1922 [114]. He achieved most of his proposed objectives. One of the most desired became a reality in 1953: the opening of the UT Best Research Institute. The historian Michael Bliss claimed that “Best distortions of the historical records seem to amount to a deliberate, unethical exercise in falsification which verges on scientific fraud. In the later years of his life Charles Best appears to have had a profound hunger for recognition, a serious ego-problem, which overwhelmed his good sense” [115].

### Best’s death (1978)

On March 31, 1978, Charles Best died after suffering a stroke. He and his wife, Margaret Mahon are buried in Pleasant Cemetery, in a grave close to that of Frederick and Henrietta Banting [26].

#### The myth of Banting and Best

Michael Bliss published in 1988 what he described as "An adventure between archives to discover documents on insulin". He interviewed Richard Landon, director of the Fisher Rare Book Room at the University of Toronto Library. He accessed confidential documentation that he described as “dynamite material”: Banting's moves to feudalize the discovery, his multiple displays of hostility towards Macleod, his excessive drinking, and episodes of verbal violence against Macleod and Collip and physical violence against Collip were reported in detail.

Reading the biography of Charles and Margaret Best, written by their son Henry, he noted his father's frequent cyclical episodes of severe depression. In several interviews with Bliss, McGill University psychiatrist Robert H. Cleghorn, well informed about the Toronto team of researchers, did not hesitate to attribute Charlest Best as a megalomaniac [116].

Bliss and other researchers have dismantled the myth of Banting and Best and have contributed to the rehabilitation of Macleod. Two articles on this subject, published in 1993 and 2013, deserve special mention [115, 117].

##### Concluding remarks

**Table:** 

(1) The European researchers G. Zülzer (1908) and N. Paulescu (1921) meet the requirements of the priority rule in the discovery of the antidiabetic hormone
(2) Socioeconomic and political factors related with the First World War and the inter-war period delayed the process of purification of the antidiabetic hormone in Europe
(3) The Canadian scientist J. B. Collip, University of Alberta, temporarily assimilated to the research team of the UT, and the American chemist and researcher G. B. Walden, employee of the pharmaceutical company Eli Lilly, were the main authors of the purification process of the antidiabetic hormone
(4) The scientific evidence allows to assert that the basic research carried out by the Department of Physiology of UT, directed by J. J. R. Macleod, in conjunction with the clinical research undertaken at the Department of Medicine of the UT, directed by D. A. Graham, made it possible in record time the successful treatment of patients suffering diabetes mellitus, that was until then a deadly disease
